# Persistent Trace Organic Contaminants Are Transformed Rapidly under Sulfate- and Fe(III)-Reducing Conditions in a Nature-Based Subsurface Water Treatment System

**DOI:** 10.1021/acs.est.3c03719

**Published:** 2023-10-19

**Authors:** Angela N. Stiegler, Aidan R. Cecchetti, Rachel C. Scholes, David L. Sedlak

**Affiliations:** † Department of Civil & Environmental Engineering, University of California, Berkeley Berkeley, California 94720, United States; ‡ Engineering Research Center (ERC) for Reinventing the Nation’s Urban Water Infrastructure (ReNUWIt), Stanford University, Stanford, California 94305, United States

**Keywords:** redox, horizontal levee, nature-based solution, micropollutants, biotransformation, anaerobic, wetland

## Abstract

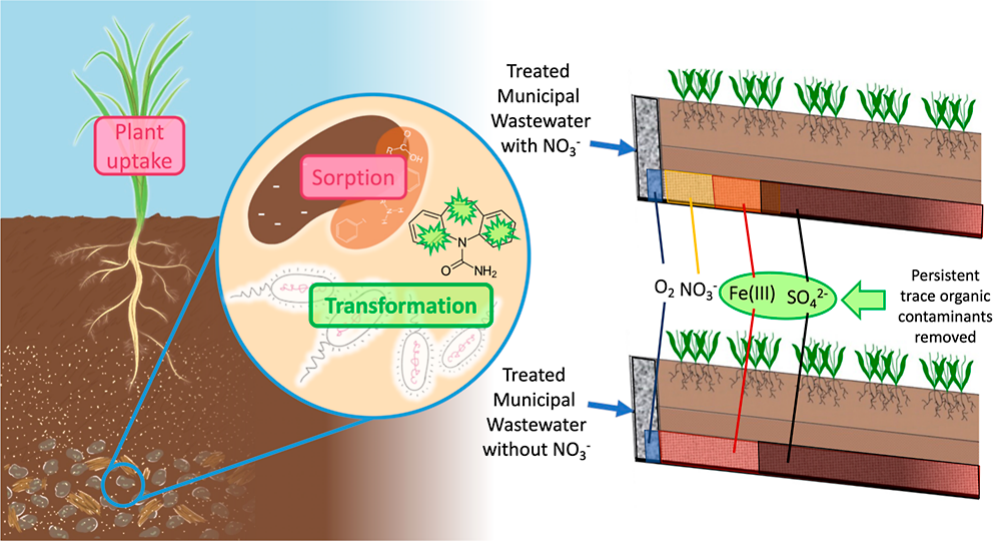

Subsurface treatment systems, such as constructed wetlands, riverbank filtration systems, and managed aquifer recharge systems, offer a low-cost means of removing trace organic contaminants from treated municipal wastewater. To assess the processes through which trace organic contaminants are removed in subsurface treatment systems, pharmaceuticals and several major metabolites were measured in porewater, sediment, and plants within a horizontal levee (i.e., a subsurface flow wetland that receives treated municipal wastewater). Concentrations of trace organic contaminants in each wetland compartment rapidly declined along the flow path. Mass balance calculations, analysis of transformation products, microcosm experiments, and one-dimensional transport modeling demonstrated that more than 60% of the contaminant removal could be attributed to transformation. Monitoring of the system with and without nitrate in the wetland inflow indicated that relatively biodegradable trace organic contaminants, such as acyclovir and metoprolol, were rapidly transformed under both operating conditions. Trace organic contaminants that are normally persistent in biological treatment systems (e.g., sulfamethoxazole and carbamazepine) were removed only when Fe(III)- and sulfate-reducing conditions were observed. Minor structural modifications to trace organic contaminants (e.g., hydroxylation) altered the pathways and extents of trace organic contaminant transformation under different redox conditions. These findings indicate that subsurface treatment systems can be designed to remove both labile and persistent trace organic contaminants via transformation if they are designed and operated in a manner that results in sulfate-and Fe(III)-reducing conditions.

## Abstract 2

Low-cost subsurface treatment systems, such as constructed wetlands, can remove persistent trace organic contaminants from water via biotransformation if they are designed to reach sulfate- and iron-reducing conditions.

## Introduction

Conventional treatment processes employed at municipal wastewater treatment plants are not designed to remove hydrophilic trace organic contaminants (i.e., micropollutants) such as pharmaceuticals, personal care products, urban use pesticides, and industrial chemicals. Therefore, these chemicals often occur in treated wastewater at concentrations that may pose risks to downstream ecosystems and wastewater-impacted drinking water supplies.^1−3^ Removal of hydrophilic contaminants that are not readily transformed in wastewater treatment plants represents a significant challenge because treatment processes that rely upon oxidation (e.g., effluent ozonation) and physical separation (e.g., activated carbon filtration) to remove trace organic contaminants can be expensive to construct, operate, and maintain.^4−6^

In the past few decades, subsurface flow treatment systems, such as constructed wetlands, riverbank filtration, and managed aquifer recharge systems have increasingly been applied as a means of removing trace organic contaminants from treated wastewater and river water.^7−9^ Although many contaminants are removed in these systems under aerobic conditions, removal of certain recalcitrant compounds, like sulfamethoxazole and carbamazepine, has only been observed to an appreciable degree under more reducing conditions in riverbank filtration systems and anaerobic membrane bioreactors.^10−15^ Anaerobic microorganisms employ different enzymes and metabolic strategies than the aerobes that dominate carbon processing during wastewater treatment (e.g., activated sludge) and may promote biotransformation of trace organic contaminants that are more slowly biotransformed under aerobic or nitrate-reducing conditions.^8,16^

Despite the potential for better performance with respect to trace organic contaminant removal, subsurface flow treatment systems have not typically been designed to reach anaerobic conditions due to a lack of labile organic carbon in the water. The addition of labile organic carbon to drive conditions to a lower redox potential is also usually avoided due to concerns about clogging and the formation of undesirable byproducts [e.g., sulfide, Fe(II)]. Therefore, it is unclear whether the intentional addition of organic carbon to subsurface treatment systems will result in a faster transformation of trace organic contaminants, especially when treatment systems are operated over extended periods.

In subsurface flow constructed wetlands, several simultaneous removal mechanisms (e.g., sorption, biotransformation, and plant uptake) can be responsible for trace organic contaminant removal. Quantifying the relative contribution of these processes could provide insight into how design and operational variables impact the performance of subsurface flow treatment wetlands. Despite the potential benefits of understanding the mechanisms responsible for trace organic contaminant removal in subsurface flow wetlands, most prior studies are limited to observations of concentrations of trace organic contaminants in water flowing into and out of full-scale systems.^17,18^

Recently, horizontal levees have been developed as a multibenefit treatment system consisting of a gradually sloped subsurface flow treatment wetland that provides sea level rise adaptation, wetland habitat, and water quality benefits. The treatment system includes a porous woodchip-amended underground treatment layer and a vegetated surface layer consisting of loamy soil and decomposing wetland plants. The woodchips and plant roots provide labile organic carbon to the wastewater effluent as it flows through the subsurface. In the first demonstration-scale horizontal levee system, which became operational in 2017, nearly all of the nitrate in municipal wastewater effluent was removed via denitrification within the first 5 m of the 45 m long treatment zone.^19−21^ After the nitrate was consumed, excess labile organic carbon supported anaerobic microbial communities, producing Fe(III)- and sulfate-reducing conditions occurring consecutively in the spatiotemporal order dictated by thermodynamics.^20^

This gradient of redox conditions provided us with a unique opportunity to assess the role that specific terminal electron acceptors [e.g., nitrate, sulfate, Fe(III)] play in the transformation of trace organic contaminants. We quantified the relative contributions of various treatment mechanisms (i.e., sorption, plant uptake, and transformation) to the removal of a diverse suite of trace organic contaminants in treated wastewater by analyzing those contaminants and common transformation products in porewater samples, sediments, and plant tissues collected at different distances from the inlet. These methods have rarely been applied to field-scale studies of constructed wetlands in the past. High resolution porewater sampling allowed us to delineate redox zones and concurrent trace organic contaminant removal with more precision than in previous field-scale research.^10−12,14,15^ Field measurements were supported by mass balance calculations, fate and transport modeling, and microcosm experiments.

## Materials and Methods

### Field Site

**Figure 1:**
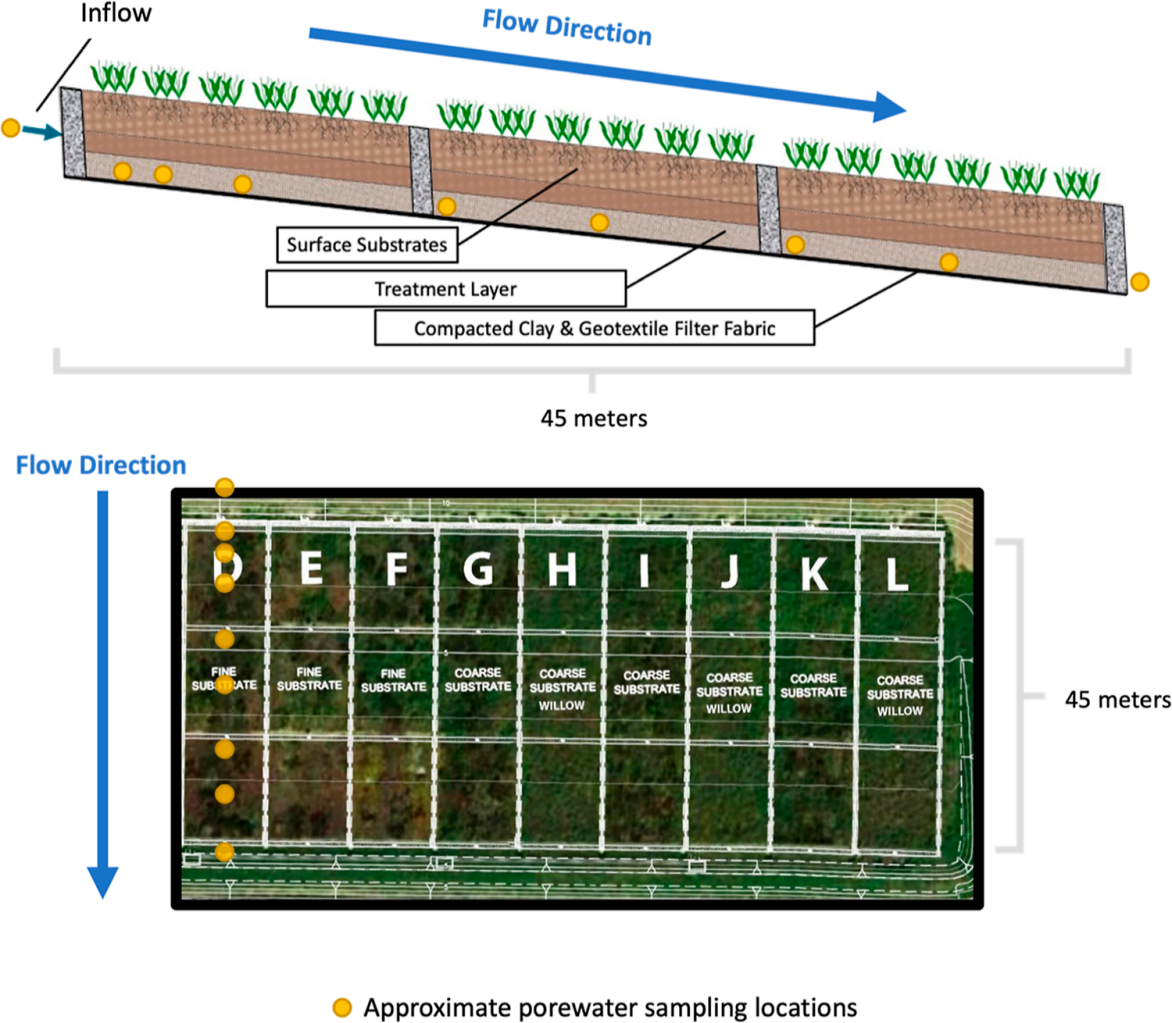
Top: Cross-section of the subsurface design of horizontal levees. Bottom: Satellite imagery of the experimental horizontal levee is overlaid by topographic contours showing parallel wetland cell configurations. Cells D–F were built with fine surface sediments. Cells G–L were built with coarser surface sediments. All cells were planted with a native plant palette typical of wet meadows, except for cells H, J, and L, which were planted with willow trees (*Salix spp.*). Sampling locations (yellow circles) were collected at 0 1.5, 2.5, 7.6, 15.8, 23, 31, 38, and 45 m.

Samples were collected from a 0.7 ha experimental horizontal levee that was described previously.^19^ Briefly, the wetland consisted of 9 hydraulically isolated, 45 m long, parallel wetland cells with treatments that varied based on surface sediment type (e.g., “fine” vs. “coarse”) and vegetation (e.g., “native meadow” vs. “willows”) (Figure 1).^19,22^ The hydraulic residence time (HRT) in the wetland cells was approximately 12–20 days. Surface sediments were underlain by a 30 cm thick treatment layer (i.e., sand, gravel, and woodchips) on top of compacted clay and a geotextile fabric liner to prevent exchange with groundwater (Figure 1).

Effluent from an activated sludge wastewater treatment plant was continuously fed into the subsurface treatment layer of the wetland cells starting in April of 2017. In August of 2020, the wastewater treatment plant was upgraded to achieve biological nutrient removal. This resulted in a decrease in the concentration of nitrogen entering the horizontal levee inflow, with nitrate dropping from approximately 30 to <1 mg N/L starting between June and August of 2021. Total nitrogen also dropped from approximately 40 to 1.5 mg N/L.

### Sampling

Wetland inflow and outflow samples were collected at approximately monthly intervals from the horizontal levee wetland cells between August 2018 and July 2019 according to methods that were described previously.^19^ Briefly, inflow and outflow samples were collected using a Masterflex E/S portable water sampler (Cole-Parmer). Inflow samples were collected twice on the same day in duplicate or triplicate from a single point prior to flowing into all 9 horizontal levee cells. Outflow samples were collected in duplicate or in triplicate from each wetland cell type. Porewater samples were collected from 5 to 7 locations along the wetland flow path (as identified in Figure 1) in all 9 wetland cells in June and July 2019 to represent summer conditions. A set of samples was collected from the same locations in three fine-textured wetland cells (i.e., Cells D–F) in March 2019 to represent winter conditions. In June 2022, samples were collected from a fine-textured wetland cell (i.e., Cell F) to assess the performance of the system after nitrate concentrations dropped in the water flowing into the horizontal levee. All porewater samples were collected using stainless steel PushPoint sediment porewater samplers (MHE Products) from a depth of approximately 0.8 m according to previously reported methods.^19^ The first 50–100 mL of extracted porewater (i.e., approximately two to four times the volume of the porewater sampling apparatus) was discarded prior to sample collection. All samples were immediately filtered through 0.7 μm glass fiber filters and stored on ice until their return to the laboratory, as described previously.^19^

In July of 2018, sediment was collected from surface sediments (depth of 0.3 m) and from the subsurface treatment layer (depth of 0.8 m) approximately 10 m from the inlet of a coarse wetland cell planted with meadow vegetation (i.e., Cell G) for use in isotherm experiments. Details about the setup of isotherm experiments are included in Section S1.3 of the Supporting Information.

### Analytical Methods

Trace organic contaminants, redox-active species, and water quality parameters were analyzed using sample-processing and analytical methods described previously.^19,23^ A list of analytes included in this research (Table S1) as well as quality control/quality assurance protocols and additional analytical details are also provided elsewhere^19,23^ and in Section S1.1 of the Supporting Information. All wetland porewater and outflow concentrations were normalized by the ratio of chloride in the sample to chloride in the wetland inflow to correct for evapotranspiration and rainfall, as described in previous publications.^19,24^ Throughout the study period, evapotranspiration accounted for approximately 40–90% of the water that flowed through the horizontal levee system. Half of the evapotranspiration occurred in the last 15 m of the system, after trace organic contaminants were removed (Figure S18).

Trace organic contaminants in freshly collected sediment used in microcosm experiments were extracted with a modified version of a procedure used previously for extractions of plant material.^23^ Details about the extraction procedure and results of spike-recovery tests for soil extractions can be found in Section S1.2 of the Supporting Information.

### Mass Balances and Estimation of the Relative Importance of Removal Mechanisms

The relative importance of the removal mechanisms in the horizontal levee: (1) sorption to sediment, (2) plant uptake, and (3) transformation reactions (i.e., biotransformation and abiotic reactions) was estimated by mass balance. Mass balances were performed by assuming that the inflow of contaminants into the wetland was balanced by the mass of contaminants exiting in wetland outflow, sorption to sediments, uptake by plants, the mass of contaminants remaining in porewater, and transformation of contaminants through microbially mediated and abiotic processes. Mass flow rates were computed using data reported elsewhere, such as inflow flow rates,^19^ annual plant biomass production,^21^ and concentrations of trace organic contaminants measured in plant tissues.^23^ Details about the mass balance calculations are provided in Section S2.1 of the Supporting Information.

Data from isotherm experiments (Figure S3) and trace organic contaminant concentrations in water samples were used in the mass balance calculations to estimate the magnitude of sorption. The magnitude of plant uptake was estimated as described previously.^23^ The magnitude of transformation reactions was quantified by mass balance, and their importance was supported by laboratory experiments designed to isolate different transformation processes, as described in Sections S4.3 and S4.4 of the Supporting Information.

Additional information about methods, such as the delineation of redox zones, estimates of hydraulic retention time, and one-dimensional transport modeling to predict contaminant breakthrough times due to sorption, can be found in Sections S2 and S3 of the Supporting Information. Pearson correlation coefficients were used to assess the strength of the linear correlation analyses. The full monitoring data set is found on Mendeley Data.^25^

## Results and Discussion

### Contaminant Removal Extent and Mechanisms

**Figure 2:**
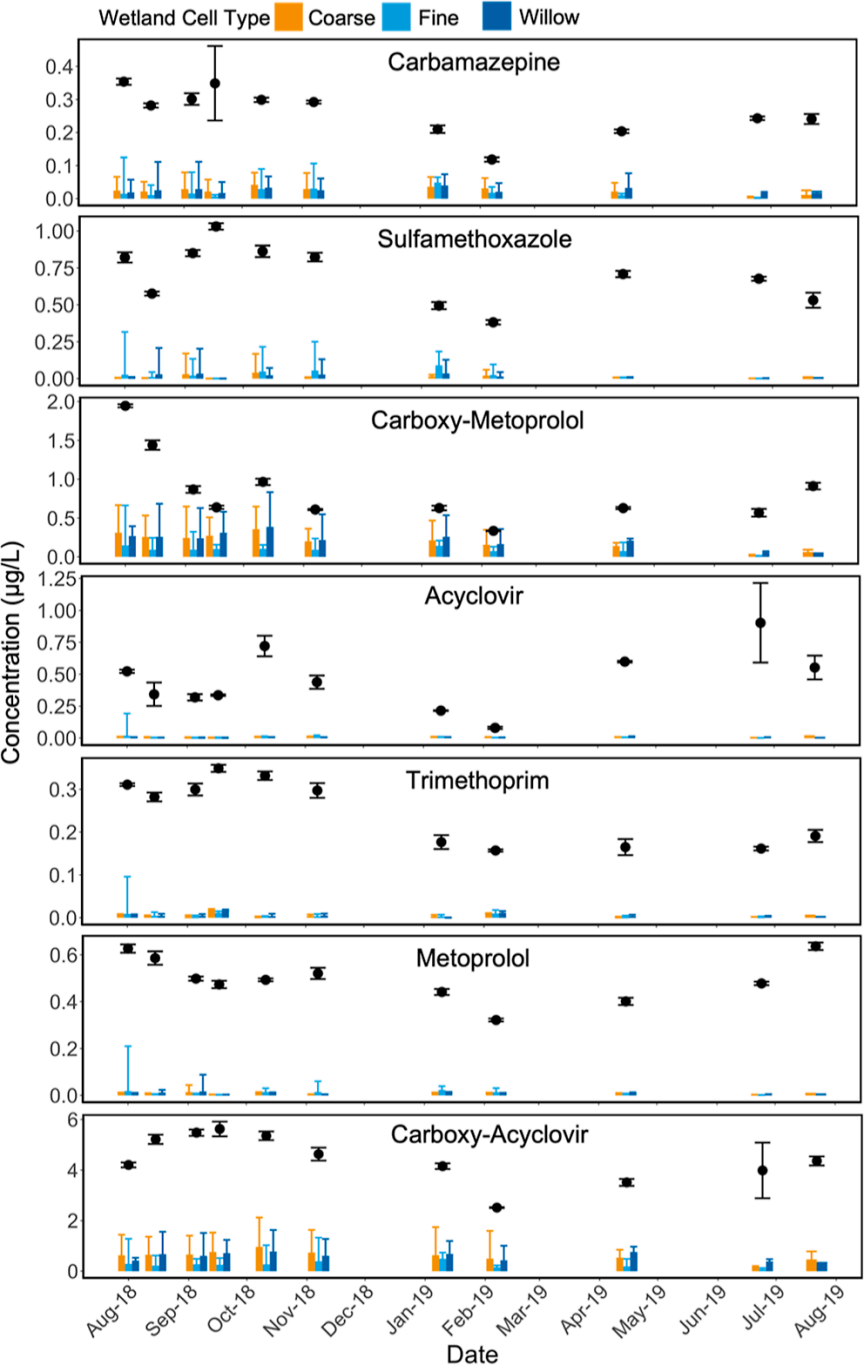
Median wetland inflow (dots) and outflow (bars) concentrations of a suite of trace organic contaminants in coarse, fine, and willow horizontal levee cells (bars, *n* = 6–9; dots, *n* = 4–6) over a 12 month monitoring period. Inflow concentrations were similar to those reported in other studies of trace organic contaminants in treated wastewater effluent.^26^

Concentrations of trace organic contaminants, including persistent compounds (e.g., carbamazepine and sulfamethoxazole), consistently decreased by over 70% in the horizontal levee in all wetland cell types (Figure 2). Carboxy-metoprolol, a metabolite of both metoprolol and atenolol that is commonly present in wastewater effluent, may have been produced within the horizontal levee and was not completely removed in the subsurface treatment system.

Concentrations of trace organic contaminants measured in the water entering the horizontal levee were as much as 50% lower in January and February of 2019. This was likely due to seasonal rainfall that increased inflow and infiltration into local sanitary sewers and caused dilution of the raw sewage flowing into the treatment plant that provided treated wastewater to the wetland system. Additional details about seasonal trace organic contaminant removal rates and the performance of different wetland cell types are presented in Section S4.2 of the Supporting Information.

**Figure 3:**
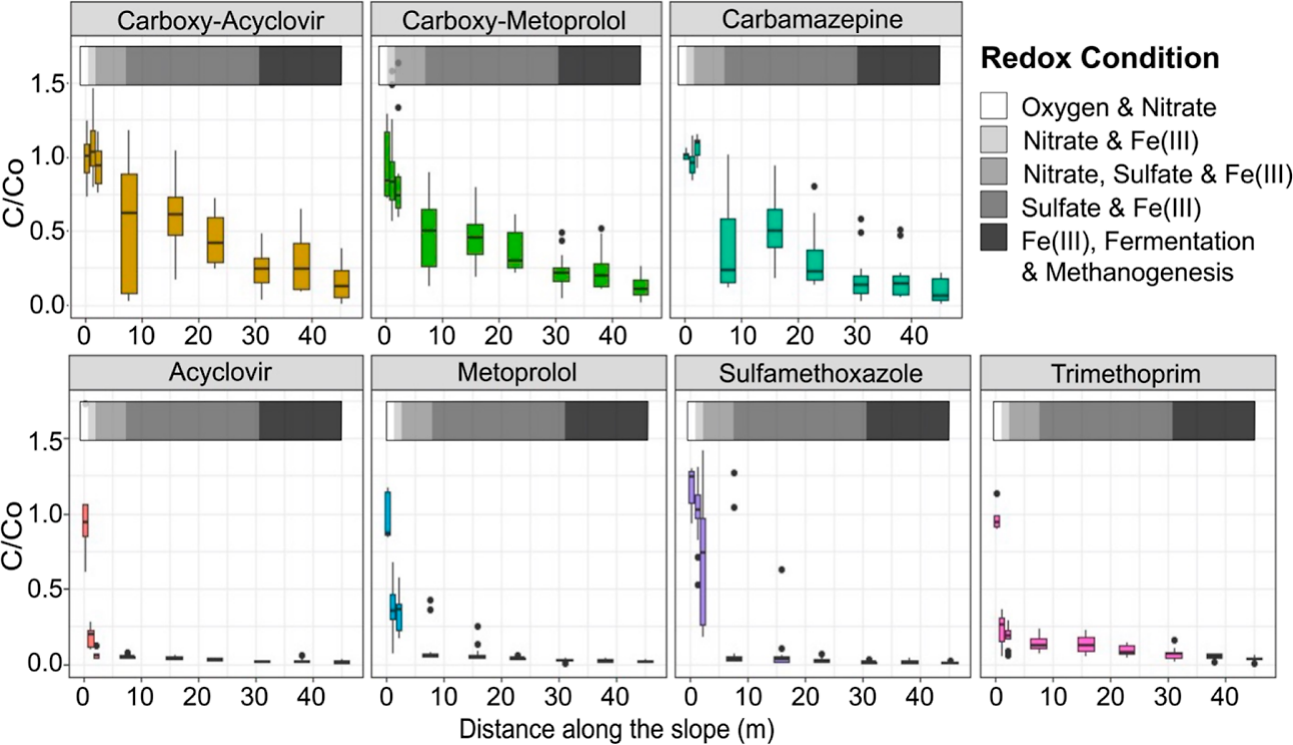
Fraction remaining of a suite of trace organic contaminants in porewater samples collected along the horizontal levee slope in all wetland cells. Samples were collected in June and July of 2019, approximately one year after most predicted sorption breakthrough times. The redox condition key refers to the horizontal bars at the top of each figure. The hydraulic residence time for the horizontal levee cells was approximately 12–20 days. (Boxplots *n* = 10–18).

The possible mechanisms of trace organic contaminant removal in the horizontal levee wetland cells included sorption to sediment, plant uptake, and transformation reactions (i.e., biotransformation and abiotic reactions). Analysis of data collected from sorption isotherm experiments (Figure S3), porewater sampling (Figure 3) conducted after expected breakthrough times predicted using one-dimensional fixed bed sorption modeling (Section S2.5 of Supporting Information), and concentrations of trace organic contaminants measured in soil and plants indicated that only a small fraction of the trace organic contaminant removal could be explained by sorption or plant uptake. For example, the concentrations of trace organic contaminants dropped along the water flow path (Figure 3) in samples collected approximately one year after most of the contaminants were predicted to have broken through the subsurface (i.e., the total operation time was 2–2.5 times the predicted breakthrough times). In addition, carbamazepine was the only monitored compound that was consistently detected in plant tissues,^23^ signifying that plant uptake and translocation was not an important removal mechanism for the other trace organic contaminants. Estimates of the extent of carbamazepine plant uptake made using concentrations of carbamazepine in plants,^23^ approximations of in-planta metabolism,^27,28^ and biomass measurements,^21^ demonstrated that plant uptake and translocation accounted for less than 12% of the mass of carbamazepine that entered the horizontal levee during the monitoring period (Figure S6, Section S2.1 for mass balance calculations).

Based on these findings, we determined that the majority (i.e., >60%) of the mass of trace organic contaminants removed in the subsurface during the monitoring period was due to transformation reactions (Figure S6). Many reactions could explain the transformation of each trace organic contaminant in the subsurface, including microbial biotransformation (e.g., anaerobic cometabolism by enzymes)^10,29−32^ and abiotic reactions involving microbially generated reactive species that are formed under anaerobic conditions [e.g., HS^–^ and Fe(II)].^30,33^ Additional observations support our conclusion that transformation reactions were the main explanation for the decreases in trace organic contaminant concentrations. For example, we would expect a relationship between porewater redox conditions and removal of trace organic contaminants (described in the next section) only if the different transformation reactions occurred under specific redox conditions. Similarly, we observed the formation of previously reported transformation products and the removal of trace organic contaminants via transformation reactions in microcosm experiments simulating the conditions observed in the subsurface wetland (microcosm results are found in Section S4.4 of the Supporting Information).

### Impact of Redox Conditions on Contaminant Removal

Redox conditions had a significant impact on the microbial communities and the concentrations of reactive chemical species [e.g., HS^–^ and Fe(II)] in the horizontal levee subsurface.^20^ Because diverse microbial communities can exhibit different capacities for contaminant removal, we expected the rates of microbially driven transformation of trace organic contaminants to exhibit spatial variations in the horizontal levee.^16,34^ The observed relationships between contaminant removal rates and the concentrations of terminal electron acceptors indicated that redox conditions were important for the trace organic contaminant transformation processes taking place in the subsurface.

Distinct spatial redox zones developed in the horizontal levee subsurface.^20^ Oxygen and nitrate, which were present in the treated effluent flowing into the wetland, served as the main terminal electron acceptors and were depleted within the first 7.5 m of the flow path (within a 2–3 day HRT), whereas sulfate was reduced between 3 and 30 m from the inlet (within a 0.8–13 day HRT), and Fe(III)-reducing conditions started at about 3 m and extended throughout the entire length of the horizontal levee due to slow microbial reductive dissolution of Fe(III)-containing minerals in the subsurface media used to construct the wetland (Figure S13).^20^ In later portions of the wetland, after sulfate was depleted, methanogenesis and fermentation likely co-occurred with Fe(III) reduction due to the abundance of methanogens and fermenting microorganisms in the subsurface^20^ and the tendency of these processes to coexist in Fe(III)-rich sediments.^35^

The spatial extent of the nitrate-reducing zone was consistent between different wetland cell types and seasons (complete within a 2–3 day HRT). In contrast, the length of the sulfate-reducing zone varied among different wetland cell types (i.e., sulfate reduction required longer residence times and distances in coarse and willow cells, 6–13 day HRT, than in fine cells, 4–7 day HRT) and sometimes overlapped with the nitrate-reducing zone in fine-textured wetland cells (Figure S13).^20^

Porewater data from all nine of the wetland cells indicated that certain trace organic contaminants (i.e., acyclovir, metoprolol, and trimethoprim) were transformed within the first 3 m of the inlet (i.e., the zone where oxygen and nitrate were typically the most energetically favorable terminal electron acceptors; Figure 3). Most of the sulfamethoxazole was transformed between 3 to 7.5 m from the inlet (0.8–3 day HRT) where redox conditions shifted from nitrate- to sulfate- and Fe(III)-reducing. Other contaminants (i.e., carbamazepine, carboxy-acyclovir, and carboxy-metoprolol) were not transformed to an appreciable extent in the first 7.5 m, but were transformed exclusively within the zone where sulfate and Fe(III) served as the most energetically favorable terminal electron acceptors.

**Figure 4:**
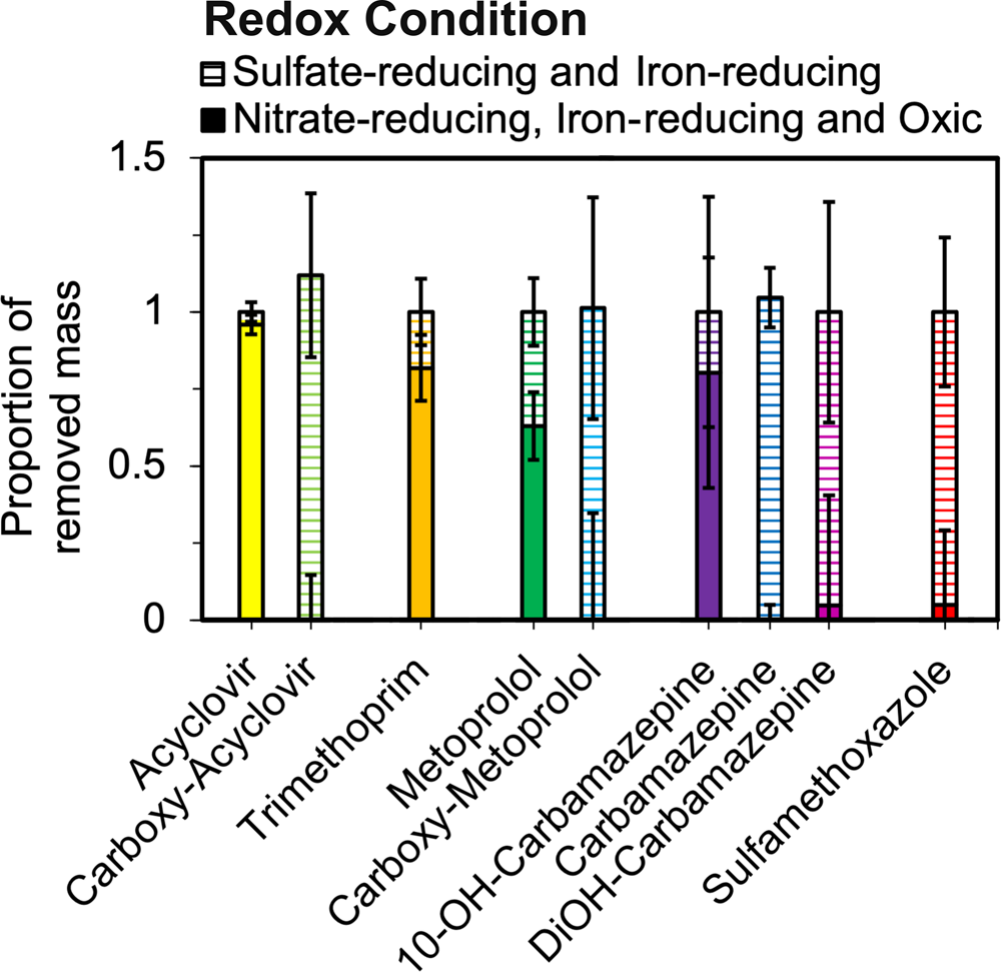
Mean mass removed in each redox zone (*n* = 10 for each contaminant) relative to the total mass removed from the inflow for a suite of trace organic contaminants. Error bars represent standard deviations and incorporate variability among the wetland cells. Wetland cells where sulfate- and nitrate-reducing conditions did not overlap were included in this analysis (wetland cells F, G, H, J, and L in Figure 1). Wetland cells with overlapping redox zones were defined as having porewater samples with more than a 10% loss of sulfate (i.e., sulfate reduction had commenced) and more than 1 mg of N/L nitrate present (i.e., denitrification was still possible). Additional details about how redox zones were defined are included in Section S3 of the Supporting Information. Some bars do not sum to unity due to small concentration increases observed in the nitrate-reducing zone.

Four of the wetland cells contained zones where nitrate and sulfate reduction overlapped, whereas five of the wetland cells had zones where nitrate- and sulfate-reducing conditions did not overlap (Figure S13). We used porewater samples from the five wetland cells without overlapping redox zones to evaluate the contribution of each redox zone to the removal of the monitored trace organic contaminants. Details about this delineation method are provided in Section S3 of the Supporting Information. Results indicated that more than 75% of the eliminated mass of acyclovir, trimethoprim, and 10-OH carbamazepine was removed in the zone with nitrate-reducing conditions. In contrast, most of the removal of persistent trace organic contaminants, such as sulfamethoxazole and carbamazepine, occurred in the zone with sulfate-reducing conditions. These results supported our hypothesis that persistent compounds could be removed if sulfate-reducing conditions are reached in horizontal levees (Figure 4).

**Figure 5:**
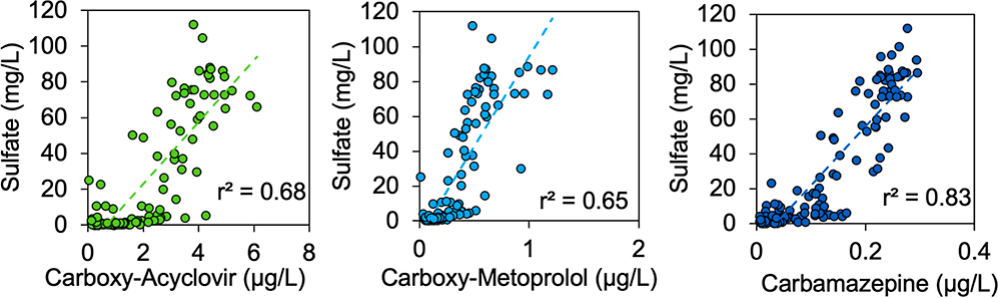
Linear correlations between concentrations of trace organic contaminants and sulfate in porewater samples collected in June–July 2019.

Strong correlations were observed between the concentrations of trace organic contaminants and dissolved terminal electron acceptors, reflecting the importance of sulfate- and Fe(III)-reducing conditions for transformation processes. Concentrations of several persistent trace organic contaminants (i.e., carbamazepine, carboxy-acyclovir, and carboxy-metoprolol) and sulfate were strongly correlated (*r*^2^ > 0.6, *p* < 0.001) in porewater samples collected from all nine wetland cells (Figure 5). Fe(III)-reduction overlapped with nitrate- and sulfate-reducing conditions in the subsurface. This implied that Fe(III)-reducing conditions may have been important to the removal of some contaminants. For example, sulfamethoxazole was removed exclusively under sulfate and Fe(III)-reducing conditions in wetland cells without overlapping nitrate- and sulfate-reducing zones, but its concentrations were also strongly linearly correlated with nitrate concentrations (Figure S16, *r*^2^ > 0.6, *p* < 0.001). Considering this and because rapid sulfamethoxazole removal has been observed under Fe(III)-reducing conditions in microcosms previously,^30^ we conclude that Fe(III)-reducing conditions likely played an important role in its transformation in the subsurface of the horizontal levee. Scatterplots for all other trace organic contaminants are included in Section S4.5 of the Supporting Information.

**Figure 6:**
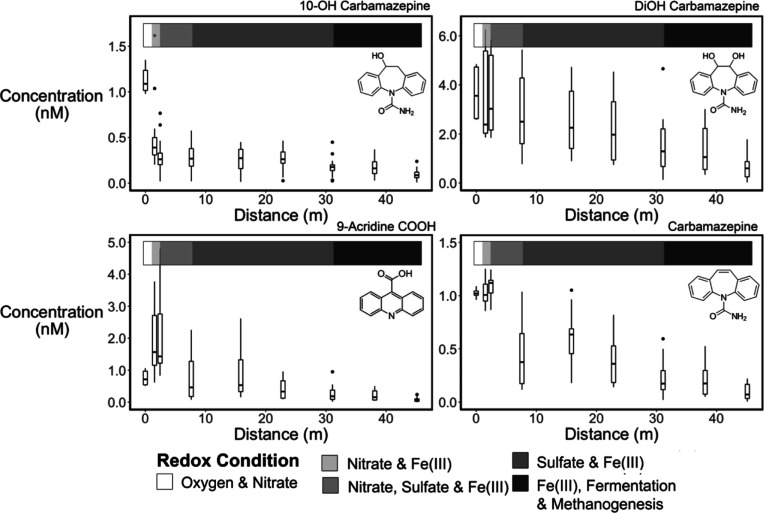
Concentrations of carbamazepine, related hydroxylated forms of carbamazepine, and their known transformation product (9-acridine carboxylic acid) in the horizontal levee subsurface. Approximate redox zones are shown in the shaded bar at the top of each plot (*n* = 4 in the first boxplot where distance is 0 m, *n* = 18 in each porewater boxplot further along the slope).

Analysis of the concentrations of carbamazepine and its metabolites in porewater collected at different locations in the wetland suggested that the rates and pathways of transformation differed greatly under different redox conditions despite structural similarities between carbamazepine and its metabolites (Figure 6). For example, concentrations of 10-OH carbamazepine decreased in the aerobic and nitrate-reducing zone as 9-acridine carboxylic acid, a transformation product that has been observed in aerobic sand filters,^36^ was formed. After 9-acridine carboxylic acid was formed, it was rapidly removed under sulfate- and Fe(III)-reducing conditions. 10-OH carbamazepine was also removed in all microcosm experiments, but 9-acridine carboxylic acid was formed only in the microcosms containing nitrate, implying that the 10-OH carbamazepine transformation pathway shifted under different redox conditions (Figure S7). In contrast to the behavior of 10-OH carbamazepine, concentrations of carbamazepine in the horizontal levee decreased exclusively under sulfate-reducing conditions (Figure 6 and S14). Dihydroxy-carbamazepine concentrations gradually decreased throughout the wetland and were not correlated to either nitrate or sulfate concentrations, suggesting that several redox conditions may have played a role in its transformation.

These results suggested that hydroxylation of the 10–11 olefinic bond in carbamazepine influenced the pathways, through which carbamazepine and its metabolites were transformed under different redox conditions (Figure S10). We suspected that transformation of carbamazepine was sensitive to modifications at the 10–11 olefinic bond because it is the electron-rich site involved in abiotic and biotic reactions of carbamazepine, such as oxidation by ozone^37^ and enzymatic metabolism in humans.^38^ However, these results and those from other studies^8,36^ suggested that predictions of metabolite fate based on parent compound behavior may be inaccurate and should be interpreted with caution.

Analysis of porewater data collected after the treatment plant that supplied water to the wetland was upgraded suggested that nitrate-reducing conditions were not essential for trace organic contaminant removal in the horizontal levee. For example, under conditions in which extremely low concentrations of nitrate (i.e., <0.25 mg N/L) entered the wetland, acyclovir, sulfamethoxazole, and metoprolol were transformed over similar distances as those observed in the presence of nitrate (Figure S17). Similar results were also observed in microcosm experiments that did not contain nitrate (Figures S8 and S9).

**Figure 7:**
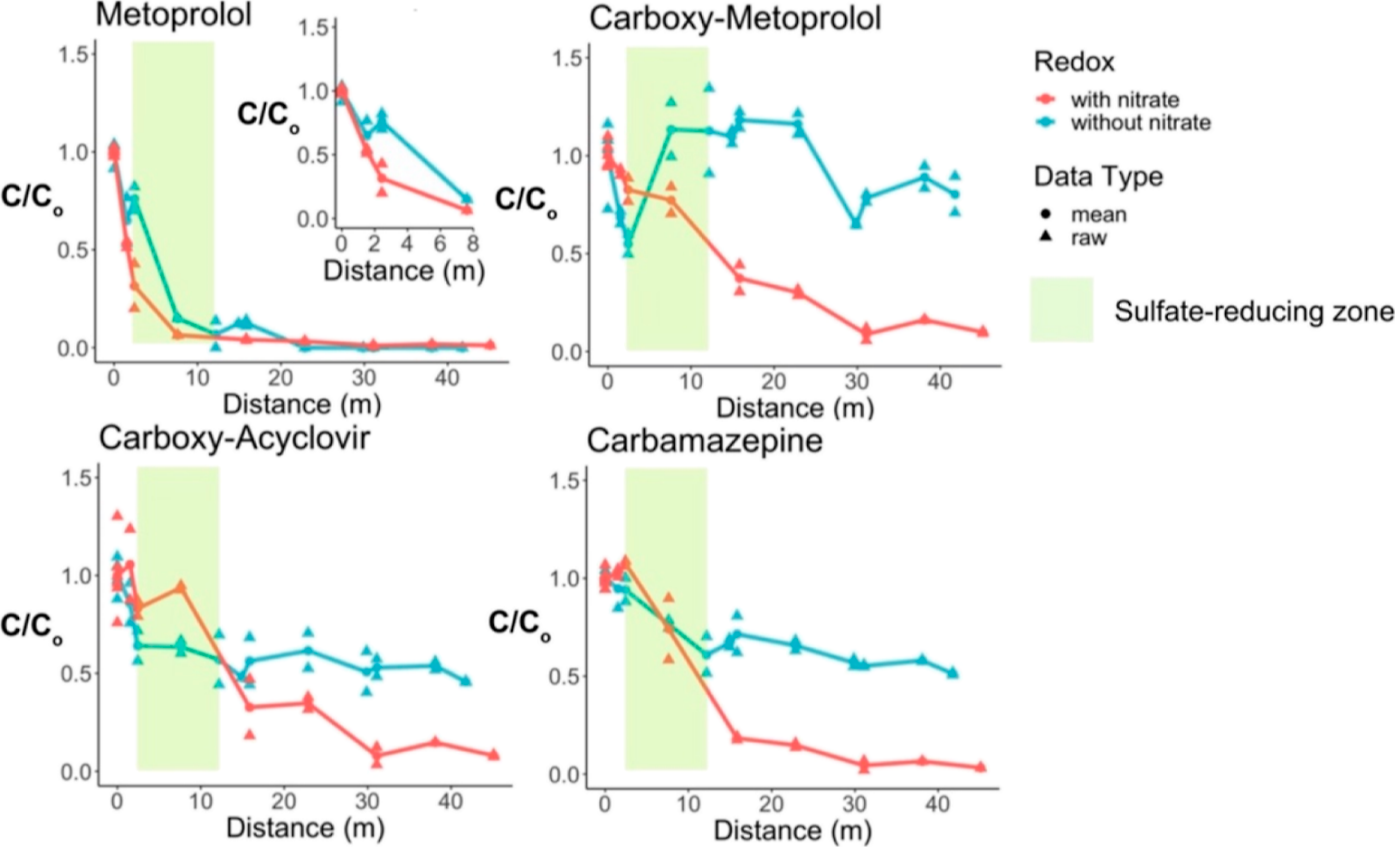
Fraction remaining of trace organic contaminants in porewater collected from the same wetland cell (i.e., Cell F) before and after wastewater treatment plant upgrades lowered nitrate concentrations in the water that flowed into the wetland. Sulfate reduction occurred over the same distance in the presence and absence of nitrate.

Interestingly, although carboxy-metoprolol was initially removed under sulfate- and Fe(III)-reducing conditions, its transformation changed after inflow nitrate concentrations dropped; concentrations of carboxy-metoprolol decreased in the first two meters before they increased to levels above the inflow concentrations after sulfate-reducing conditions were reached (Figure 7). Carboxy-metoprolol was slowly removed over the remainder of the wetland where Fe(III)-reducing conditions prevailed. These results could be related to differences in the transformation of precursors to carboxy-metoprolol (e.g., metoprolol and atenolol) in the absence of nitrate. For example, prior to the wastewater treatment plant upgrades, metoprolol was removed in the nitrate-reducing zone, before sulfate-reducing conditions were reached. After upgrades, metoprolol was removed under sulfate-reducing conditions, which coincided with the observed increases in carboxy-metoprolol concentrations. Future research into the anaerobic transformation pathways of metoprolol as well as atenolol is needed to gain insight into the transformation process because the formation of carboxy-metoprolol has not been previously reported under anaerobic conditions.

After treatment plant upgrades, lower sulfate concentrations in the inflow to the wetland may have led to less removal of some trace organic contaminants because the rates of microbial sulfate reduction are sulfate-limited below concentrations of approximately 100 mg/L.^39,40^ The concentration of sulfate in the inflow to the horizontal levee when porewater samples were collected was approximately 70 mg/L in 2019 (i.e., pre-upgrades) versus approximately 40 mg/L in 2022 (i.e., post-upgrades). It is unlikely that treatment plant upgrades caused this decline because biological nutrient removal processes do not typically remove appreciable amounts of sulfate.^41^ Variability in the concentrations of sulfate in the wastewater effluent was also observed prior to the treatment plant upgrades (Figure S11) and may have been caused by routine changes in the source water used to supply drinking water to the community served by the wastewater treatment plant (Figure S12). A lack of nitrate flowing into the wetland also could have lowered internal sources of sulfate in the subsurface because autotrophic sulfide-driven denitrification had previously regenerated sulfate during the cooler seasons (i.e., December–March) in the subsurface.^20^ Regardless, the lower concentration of sulfate in the water that flowed through the system at the time of sampling likely limited the activity of sulfate-reducing microbial communities in the subsurface and may have been responsible for the decrease in rates of transformation of compounds that were previously transformed under sulfate-reducing conditions. For example, carbamazepine was not completely transformed after treatment plant upgrades (e.g., approximately 50% removal post-upgrades versus >80% removal pre-upgrades). Because carbamazepine removal exclusively took place in the sulfate-reducing zone under both sets of conditions, carbamazepine concentrations stabilized after sulfate was depleted from the subsurface. This finding strongly supports the hypothesis that sulfate-reducing conditions were important to the transformation of carbamazepine and other persistent trace organic contaminants.

Changes in the nature of the organic carbon in the treated effluent after treatment plant upgrades could have impacted the horizontal levee treatment performance^42^ because carbon sources can alter microbial transformation of trace organic contaminants in subsurface treatment systems. For example, low concentrations of readily biodegradable dissolved organic carbon or the presence of more persistent forms of carbon (e.g., lignocellulose, organic carbon remaining after wastewater is subjected to microbial processes) elicited the use of different enzymes by microorganisms and led to faster rates of trace organic contaminant transformation in other subsurface systems.^43−46^ In the horizontal levee, although the concentration of total organic carbon in the wetland inflow was consistent before and after treatment plant upgrades (i.e., approximately 8 mg of C/L), the carbon that persisted in the treated effluent after plant upgrades was exposed to anoxic and aerobic treatment processes. In contrast, prior to upgrades, the wastewater-derived carbon was only exposed to aerobic conditions during treatment. Similarly, the nature and supply of organic carbon from woodchips and/or plant roots likely changed over the course of the monitoring period,^47^ which could have contributed to performance differences. These changes could have led to the removal or transformation of carbon sources that were important for microbial communities that removed trace organic contaminants under sulfate- and Fe(III)-reducing conditions in the horizontal levee subsurface. This may explain the apparent persistence of carboxy-acyclovir in the sulfate-reducing zone following treatment plant upgrades (Figure 7). In the future, research is needed to elucidate the role of the source of carbon in the measured performance differences.

### Comparison of Trace Organic Removal in Different Types of Treatment Systems

**Figure 8:**
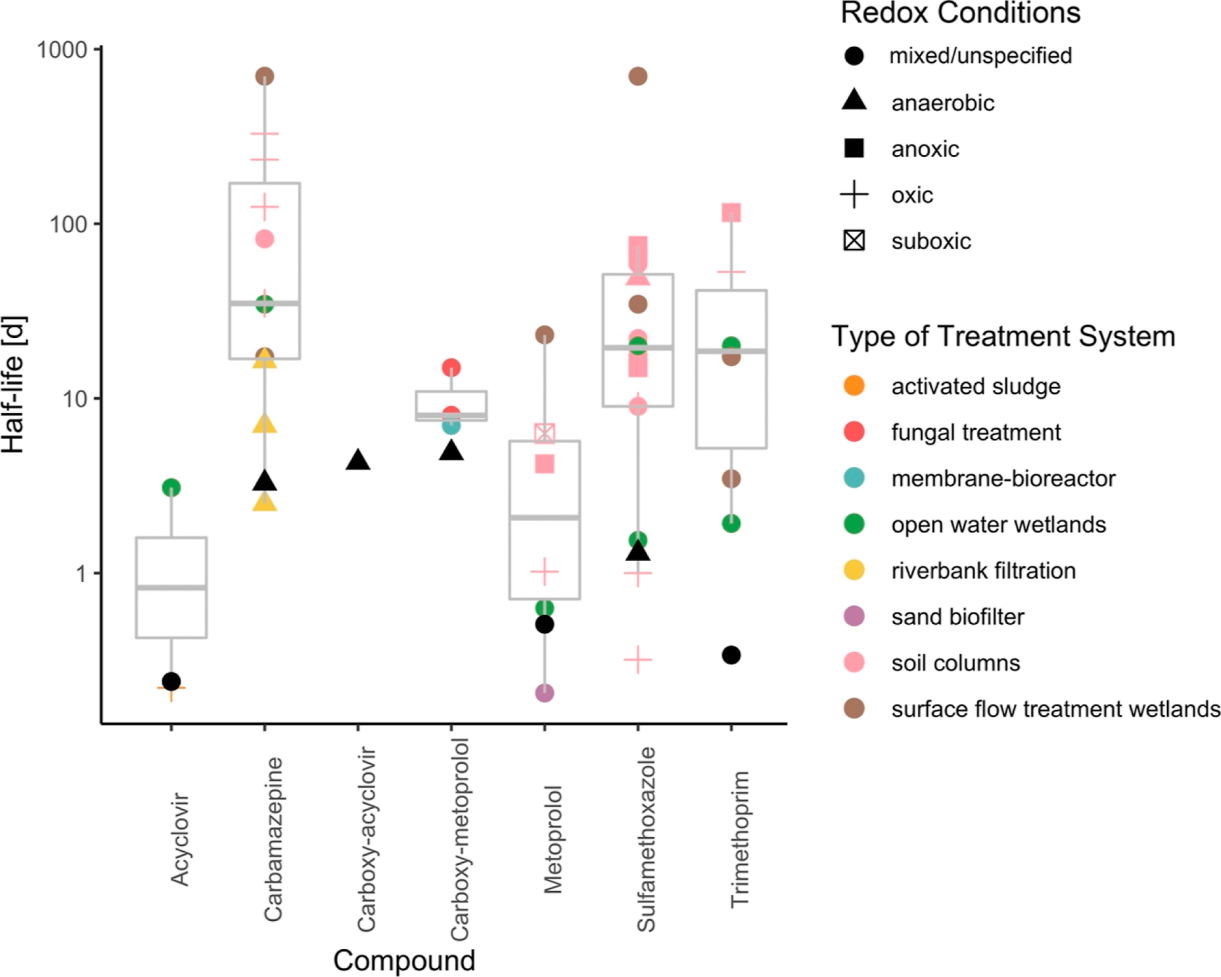
Half-lives for the monitored trace organic contaminants in the horizontal levee prior to upgrades made to the upstream wastewater treatment plant (black triangles and circles) compared to those in other treatment systems (colored symbols). The half-lives for the other treatment systems are summarized in the gray box plots.^11,14,48−57^ After treatment plant upgrades, the half-lives for sulfamethoxazole, metoprolol, and acyclovir did not change. Removal of carbamazepine, carboxy-acyclovir, and carboxy-metoprolol varied spatially after treatment plant upgrades, which limited the relevance of half-life predictions over the entire horizontal levee system under these conditions.

To compare the performance of other treatment systems to that of horizontal levees operated when sufficient sulfate is supplied to the subsurface, we used porewater concentration data collected prior to upgrades to the wastewater treatment plant and hydraulic residence times to estimate half-lives for various trace organic contaminants (see Section S2.3 of the Supporting Information for details). Shorter half-lives could reduce the size needed for treatment in constructed wetlands, which is often an important constraint for their application in areas with limited land availability. Prior to the upgrade of the wastewater treatment plant, the half-lives for trace organic contaminants that are typically persistent in biological treatment systems (e.g., carbamazepine and sulfamethoxazole) were less than 10 days in the horizontal levee, which is considerably shorter than the median values reported for other treatment systems (Figure 8). The only trace organic contaminant management approaches that had comparable half-lives were carbamazepine removal in an anaerobic riverbank filtration system^11^ and sulfamethoxazole removal from a managed aquifer recharge system that treated water with a relatively low concentration of biodegradable dissolved organic carbon.^48^ In all cases where rapid carbamazepine removal was previously reported (i.e., half-life less than 10 days), unspecified anaerobic conditions were observed, but sulfate-reducing conditions were plausible, further supporting our conclusions that sulfate-reducing conditions enhance rates of carbamazepine removal.

The half-lives of acyclovir and metoprolol, two compounds that are relatively biodegradable under aerobic conditions, were less than 1 day in the horizontal levee, which is similar to or faster than those reported for other aerobic treatment systems (Figure 8). This is significant because aerobic or anoxic conditions can result in faster transformation of certain types of labile organic contaminants^58−60^ due to higher microbial growth rates and greater energy yields from the use of oxygen and nitrate as terminal electron acceptors. The horizontal levee could remove these compounds if either oxygen or nitrate was available in the inflow to the subsurface.

This half-life analysis serves as a means of comparing the horizontal levee performance to those of other systems. Our analysis demonstrates that horizontal levees remove contaminants more quickly than most other treatment systems, which is likely driven by the design of the subsurface [i.e., presence of sulfate- and Fe(III)-reducing conditions]. However, half-lives should not be used in isolation to predict the performance of subsurface treatment systems because they cannot capture the complexity of the removal processes, and half-lives are not applicable to wastewaters with differing compositions (e.g., different concentrations of terminal electron acceptors) or upstream treatment processes.^48^

### Environmental Implications

The horizontal levee rapidly removed persistent trace organic contaminants via transformation in the subsurface, likely due to the development of sulfate- and Fe(III)-reducing conditions and sufficiently long hydraulic retention times (e.g., several weeks). Such sulfate and Fe(III)-reducing conditions typically require extended hydraulic residence times to develop in riverbank filtration or managed aquifer recharge systems due to limited amounts of assimilable carbon in treated wastewater or river water. For example, production wells that exhibit Fe(III)-reducing conditions during riverbank filtration often exhibit hydraulic residence times on the order of months or longer.^14^ Furthermore, there is hesitation to amend subsurface treatment systems with assimilable organic carbon due to concerns about clogging, mercury methylation, and sulfide or Fe(II) production. However, horizontal levees, which are used to remove contaminants from treated effluent, may not create these issues due to a lack of clogging or hydraulic performance issues observed in this research, low levels of mercury in treated wastewater, and the formation of minerals in the horizontal levee subsurface, which limited the concentrations of dissolved Fe(II) and sulfide.^20^

Horizontal levees and other subsurface flow treatment wetlands could be particularly well-suited for trace organic contaminant removal via transformation reactions taking place under sulfate- and Fe(III)-reducing conditions because they contain plant roots and exudates from plants growing in the treatment system that continuously release carbon to the subsurface.^61^ Sources of carbon originating from plants may provide a means of maintaining sulfate- and Fe(III)-reducing conditions for extended periods of operation without the need to supplement organic carbon.^20^ However, initial organic carbon supplements (e.g., woodchips) such as those employed in the horizontal levee could last for decades^20^ and could ensure sulfate- and Fe(III)-reducing conditions exist prior to the establishment of plant roots. In addition, the availability of Fe(III) minerals and sulfate as terminal electron acceptors should be considered when subsurface flow treatment systems that are intended to remove persistent trace organic contaminants from water.

Although our findings suggest that sulfate reduction was important to the removal of certain recalcitrant trace organic contaminants such as carbamazepine, we are unable to attribute removal exclusively to sulfate-reducing microorganisms because they often coexist with or rely upon other anaerobic microorganisms in sediments and soils. For example, sulfate-reducers often form close associations with fermenters or microorganisms that can break down solid forms of carbon, such as those found in woodchips.^62−64^ The microbial community in the horizontal levee is comprised of a complex consortia of interdependent and/or competing organisms,^20^ suggesting that many members of the microbial community could be responsible for trace organic contaminant transformation. Research into the structure, function, and activity of the microbial communities in anaerobic subsurface treatment systems and their links to contaminant removal is needed to identify the microbial community responsible for trace organic contaminant removal in the horizontal levee.

In addition to enzymatic reactions, microorganisms often generate reactive species as a byproduct of their metabolism that could transform trace organic contaminants under sulfate- and Fe(III)-reducing conditions. For example, sulfamethoxazole undergoes ring-cleavage through reactions with Fe(II) adsorbed onto Fe(III)-containing minerals (e.g., goethite) which is present during microbially induced reductive dissolution.^30^ Although the concentration of dissolved sulfide species are likely to be low in the horizontal levee due to excess Fe(II), sulfide species (e.g., HS^–^, polysulfides) could act as potent nucleophiles which react with organic compounds containing electron-deficient moieties in Fe(III)-deficient sediment.^33^ Regardless, anaerobic trace organic contaminant transformation pathways via biotic and microbially induced abiotic processes remain understudied, limiting our ability to predict the mechanisms and products of contaminant transformation in this field study. Future research should target the transformation pathways, kinetics, and product identities under sulfate- and Fe(III)-reducing redox conditions.

The horizontal levee rapidly removed a suite of trace organic contaminants with a wide range of previously reported biodegradability. Trace organic contaminant removal was achieved through transformation reactions under aerobic, anoxic, and anaerobic (e.g., sulfate- and Fe(III)-reducing) conditions. However, similar removal was achieved for many contaminants both with and without nitrate available as a terminal electron acceptor, demonstrating that nitrate reduction was not essential to the removal of trace organic contaminants. This is an important finding because nitrate is not present in sufficient quantities in all water sources that might be treated in a horizontal levee. For example, horizontal levees are intended to be deployed in coastal regions where wastewater treatment plants are frequently required to comply with stringent nitrogen discharge limits to prevent algal blooms and eutrophication. Managers of wastewater treatment plants equipped with nutrient removal systems that discharge very low concentrations of nitrate (i.e., <1 mg N/L) may still be interested in the removal of trace organic contaminants. Due to their ability to remove numerous trace organic contaminants and nitrate, carbon-amended subsurface treatment systems like the horizontal levee may soon find wide application as multibenefit nature-based solutions to environmental challenges.

## Supporting Information Available

Analytical methods, methods used to calculate a variety of study components (i.e., mass balances, contaminant half-lives, one-dimensional fixed bed breakthrough modeling, and estimates of hydraulic retention time); delineation of various redox conditions in the wetland; and sorption isotherms, seasonal performance, mass balances, microcosm experiments, and other additional details about the field site (PDF)
