# Supplementary material for: Persistent Trace Organic Contaminants Are Transformed Rapidly under Sulfate- and Fe(III)-Reducing Conditions in a Nature-Based Subsurface Water Treatment System
Source: Environ Sci Technol. 2023 Oct 19;57(43):16616–27. doi: 10.1021/acs.est.3c03719 (PMC10620999; doi:10.1021/acs.est.3c03719)
Supplement: Supplementary file 1 — es3c03719_si_001.pdf [file es3c03719_si_001.pdf]

Supplementary Information for

Persistent Trace Organic Contaminants are Transformed Rapidly Under Sulfate and Fe(III)-Reducing Conditions in a Nature-Based Subsurface Water Treatment System

Angela N. Stiegler[1,2], Aidan R. Cecchetti[1,2,3], Rachel C. Scholes[1,2,4] and David L. Sedlak*[1,2]

[1]Department of Civil & Environmental Engineering,
University of California, Berkeley
Berkeley, CA 94720, United States

[2]Engineering Research Center (ERC) for
Reinventing the Nation's Urban Water Infrastructure (ReNUWIt)

[3]Present address: Division of Drinking Water, State Water Resources Control Board (SWRCB),
California Environmental Protection Agency (CalEPA)
Richmond, CA 94804, United States

[4]Present address: Department of Civil Engineering,
University of British Columbia,
Vancouver, British Columbia, V6T 1Z4, Canada

Submitted to: Environmental Science & Technology

September 4, 2023

*Corresponding author email: sedlak@berkeley.edu

37 Pages, 22 Figures, 6 Tables

Table of Contents

S-1 Analytical Methods S3

S-1.1 Additional Analytical Details S3
S-1.2 Sediment Extraction Methods and Recovery Experiments S5
S-1.3 Isotherm Methods S6

S-2 Calculations S7

S-2.1 Mass balance calculations S7
S-2.1.1 Plant Uptake Scenarios S10
S-2.2 Estimates of HRT S11
S-2.3 Contaminant Half-Lives S12
S-2.4 Methods Used to Calculate Distribution Coefficients S12
S-2.5 One-Dimensional Fixed Bed Sorption Modeling and Breakthrough Times S13

S-3 Redox Zonation S14

S-4 Supplementary Results S14

S-4.1 Sorption Isotherm Results S14
S-4.2 Impact of Wetland Cell Type and Temperature on Performance S15
S-4.3 Mass Balance Results S17
S-4.4 Microcosm Experiments S19
S-4.5 Other Supplemental Results S25

S-5 Photos of the Field Site S32

S-6 References S35

List of Figures and Tables

Figure S1 Recoveries for monitored trace organic contaminants S5
Figure S2 RO Concentrate tracer S11
Figure S3 Isotherm results for the monitored trace organic contaminants.................................S14
Figure S4 Concentrations of trace organic contaminants versus distance in horizontal levee....S16
Figure S5 Concentrations of carbamazepine and metoprolol in the horizontal levee................S17
Figure S6 Mass balance...S18
Figure S7 9-Acridine-COOH formation...S22
Figure S8 Nitrate-reducing microcosms...S23
Figure S9 Sulfate-reducing microcosms S24
Figure S10 Redox dependent removal of carbamazepine and related compounds S25
Figure S11 Inlet sulfate concentrations over the monitoring period. S25
Figure S12 Sulfate concentrations in drinking water supplies S26
Figure S13 Redox conditions in all three wetland cell types S27
Figure S14 Mean concentrations of carbamazepine (dots) and sulfate (triangles). S28
Figure S15 Scatter plots showing trace organic contaminant concentrations and sulfate...........S29
Figure S16 Scatterplots showing trace organic contaminant concentrations and nitrate S30
Figure S17 Results from sampling before and after treatment plant upgrades S32
Figure S18 Evapotranspiration results……………………………………………………….S32
Figure S19 Horizontal levee in January 2017 view from the effluent sampling wells S32
Figure S20 Horizontal levee in October 2017 - view from the effluent sampling wells............S33
Figure S21 Horizontal levee in winter of 2022 (left) and summer of 2021 (right) S33
Figure S22 Wildlife use of the horizontal levee field site. S34

Table S1 Analyte List...S3
Table S2 Physicochemical properties of the monitored trace organic contaminants S4
Table S3 Plant uptake scenarios S10
Table S4 Summary of K_D values derived from isotherms, microcosms, and field results..........S15
Table S5 Experimental treatments and corresponding shorthand used for labels in Table S6 ...S20
Table S6 Sampling plan for the microcosm experiments..S21

S-1 Analytical Methods

S-1.1 Additional Analytical Details

An analyte list and spike-recoveries for soil extractions can be found in this section.

Table S1 Analyte List

Analyte type	Analyte	Instrumentation and Methods
Anions	Cl^-	Ion Chromatography (Dionex DX-120)<br><br>Anions were analyzed on a Dionex IonPac AS23 column according to U.S. EPA Method 300.0<br><br>Cations were analyzed according to previously described methods[1] with minor modifications by using a 3.0 mM methanesulfonic acid eluent and a Dionex IonPac CS16 column
	NO_3^-	
	NO_2^-	
	SO_4^{2-}	
	Br^-	
Cations	NH_4^+	
	Na^+	
	Mg^{2+}	
	Ca^{2+}	
	K^+	
	Li^+	
Organic Carbon	TOC	Shimadzu TOC analyzer with an attached TN-1 unit[2]
Nitrogen	TN	
Metals	Fe	Inductively Coupled Plasma-Mass Spectrometry[2] (Agilent 7700)
	Mn	
Trace Organic Contaminants	Acyclovir	Liquid Chromatography Mass Spectrometry[3–6] (Agilent 1200 series HPLC coupled to an Agilent 6460 triple quadrupole mass spectrometer)
Metabolite	Carboxy-Acyclovir	
	Metoprolol	
Metabolite	Carboxy-Metoprolol	
	Trimethoprim	
	Sulfamethoxazole	
	Carbamazepine	
Metabolite	10-OH-Carbamazepine	
Metabolite	DiOH-Carbamazepine	
Metabolite	9-Acridine-COOH	
General Water Quality Parameters	pH	Myron Ultrameter II
	Electrical Conductivity	
	Dissolved Oxygen	YSI ProODO Dissolved Oxygen Probe
	Temperature	

Table S2 Physicochemical properties of the monitored trace organic contaminants. All values were obtained from PubChem. Values with an * are estimates, values without an * are from laboratory measurements reported in the literature.

Compound Name	Compound Structure (At pH 7.5)	pK_a	Charge at pH 7.5	$LogK_{ow}$	$LogD_{ow}$ @pH 7.5
Acyclovir	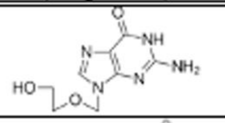	2.27<br>9.25	0	-1.56	-1.56
Carboxy-Acyclovir	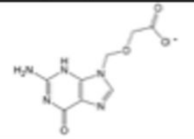	~2.27*<br>~4*<br>~9.25*	-1	<-1.56*	-6.7
Carbamazepine	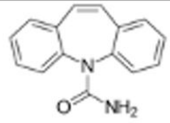	NA	0	2.45	2.45
10-OH Carbamazepine	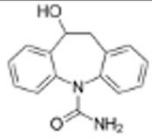	NA	0	1.4*	1.4
10-11, Dihydroxy Carbamazepine	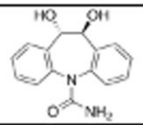	NA	0	0.3*	0.3
9-Acridine carboxylic acid	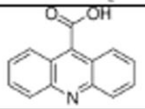	0.7*	-1	3.1*	-3.7
Sulfamethoxazole	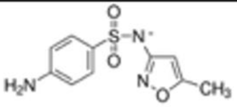	1.6<br>5.7	-1	0.89	-1.2
Metoprolol	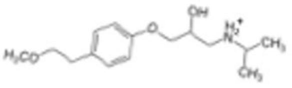	9.7	+1	1.88	-0.3
Carboxy-metoprolol	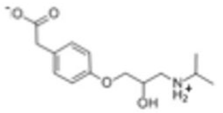	~4*<br>~9.7*	0, zwitterion	-1.5*	-5.1
Trimethoprim	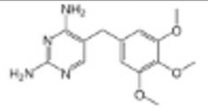	7.12	0	0.91	0.78

S-1.2 Sediment Extraction Methods and Recovery Experiments

Trace organic contaminants were extracted from 5 g of homogenized wet sediment using 12 mL of a 3:5 methanol:Milli-Q water solution via sonication and shaking for 30 minutes each. The process was repeated with 7.5 mL of a 1:1 methanol:Milli-Q water solution and combined extracts were filtered through a 0.2-µm polyethersulfone membrane prior to analysis via LCMS.

Recovery experiments were used to validate the extraction method. Briefly, an aqueous mixture containing approximately 5.8 ng of each trace organic contaminant was spiked into 5 g of moist soil in a 15 mL centrifuge tube. The spiked soil sample was mixed and allowed to equilibrate for approximately 1 hour prior to extraction. Spiked and unspiked samples were extracted to calculate recoveries. All recoveries were calculated using concentrations on a dry weight basis to account for differences in moisture content among soil samples. Results of the recovery experiments can be found in Figure S1.

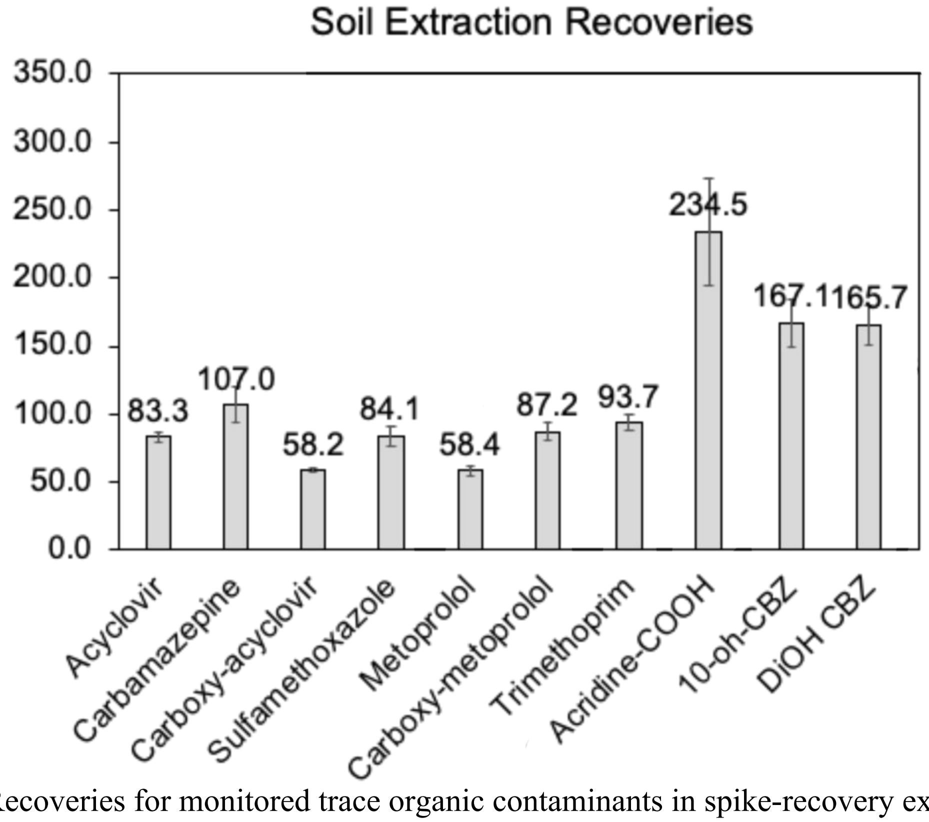

Figure S1 Recoveries for monitored trace organic contaminants in spike-recovery experiments (n =3).

Carbamazepine metabolites (Acridine-COOH, 10-OH-CBZ, and DiOH CBZ) may have had elevated recoveries relative to the other trace organic contaminants due to the use of carbamazepine-d10 as an internal standard for the quantification of those compounds. All other compounds had a corresponding stable-isotope labeled version that was used as the internal standard.

S-1.3 Isotherm Methods

Gravel larger than 0.5 cm was removed from sediment collected for isotherm experiments and the remaining sediment was allowed to air-dry at room temperature for at least 48 hours. After homogenization, 5 g of air-dried sediment was sub-sampled into 50-mL centrifuge tubes and 25 mL of an aqueous solution containing 10 mM $CaCl_2$ and increasing concentrations of trace organic contaminants was added to each tube. Trace organic contaminants were spiked into the liquid phase by adding them from an aqueous solution containing all the contaminants in a mixture. The centrifuge tubes containing sediment and water were placed on a rotary shaker and allowed to equilibrate for 24 hours prior to sample collection. Isotherms, which were run in triplicate, were conducted at concentrations that extended beyond the concentrations entering the wetland for most compounds. Distribution coefficients were compared to field-derived and microcosm-derived distribution coefficients calculated by pairing porewater concentrations with extractable sediment concentrations.

S-2 Calculations

S-2.1 Mass balance calculations

The following equation was used to describe the mass balance in the wetland over the course of the wetland operation.

$$m_{in} = m_{out} + m_{porewater} + m_{sorbed} + m_{roots} + m_{plants} + m_{transformed}$$

S.1

Where *m* is the mass in each wetland compartment over the full operation of the wetland. $m_{porewater}$ and m_{sorbed} are both storage terms that do not change during the monitoring period because porewater concentrations remained constant over the monitoring period. Therefore, they could be excluded from the mass balance during the monitoring period (i.e., the contaminants did not accumulate in the sediment or porewater over the monitoring period). However, to provide a conservative estimate of the fraction of contaminants removed via other mechanisms, which make up the remainder of the mass that entered the wetland, we assumed that all of the mass of contaminants quantified in each of these compartments ($m_{porewater}$ and m_{sorbed}) entered the wetland during the monitoring period, in the absence of sufficient inflow concentration data prior to the monitoring period. The mass that entered the wetland was quantified using flowrates averaged over entire the monitoring period and inflow concentrations averaged over each observation interval:

$$m_{in} = \int C_{in}(t)Q_{in}(t)dt = (\sum \overline{C_{in,\,\Delta t}}\,\Delta t\,)\,\overline{Q_{in}}$$

S.2

Where m_{in} is the mass of a trace organic contaminant entering the wetland over the entire monitoring period. The average inflow concentration over each monitoring date time interval time interval Δt, was $\overline{C_{in,\,\Delta t}}$, and the average inflow flowrate was $\overline{Q_{in}}$. The mass that exited the wetland was quantified by averaging outlet concentrations over the entire monitoring period due to minimal variability in outlet concentrations.

$$m_{out} = \int C_{out}(t)Q_{out}(t)dt = \overline{C_{out}}\,\overline{Q_{out}}\,\Delta t$$

S.3

Where m_{out} is the mass of a trace organic contaminants exiting the wetland over the time interval Δt at an average outflow concentration, $\overline{C_{out}}$, and outflow flowrate $\overline{Q_{out}}$. $\overline{Q_{out}}$ was approximated by accounting for evapotranspiration through increases in the concentration of the conservative ion, Cl^-.

$$Q_{out} = Q_{in}\frac{[Cl]_{in}}{[Cl]_{out}}$$

S.4

Therefore:

$$m_{out} = \overline{C_{out}}\frac{\overline{[Cl]_{in}}}{[Cl]_{out}}\overline{Q_{in}}\,\Delta t$$

S.5

The mass of contaminants sorbed to soil particles, m_{sorbed}, was calculated using isotherm K_D values and porewater concentrations, $C_{pw}(x)$. Because porewater concentrations varied spatially across the wetland, concentrations were integrated over the length of the wetland:

$$m_{sorbed} = K_D\,\rho_b\,A\int C_{pw}(x)dx = K_D\,\rho_b\,A\sum\overline{C_{pw}}\,\Delta x$$

S.6

A uniform bulk density, ρ_b, of 1.5 g cm^{-3}, and cross sectional area, A, equal to the width of each wetland cell (10^4 cm) times the total depth (10^2 cm), was used to provide a maximum estimate of sorption in the subsurface.

The mass of contaminants in porewater, $m_{porewater}$, was approximated similarly, assuming a porosity, ϕ, of 0.4.

$$m_{porewater} = \phi\,A\int C_{pw}(x)dx = \phi\,A\sum\overline{C_{pw}}\,\Delta x$$

S.7

The mass of contaminants in plants, m_{plants}, taken up during the monitoring period, Δt, was approximated using the following equation because plant biomass and concentrations in plants varied over time and space.

$$m_{plants} = w\iint C_{plants}(x)B_{shoots}(t)dtdx = B_{shoots}w\Delta t\sum\overline{C_{plants}}\,\Delta x$$

S.8

Where w, is the width of each wetland cell (10 m), $C_{plants}(x)$, is the total concentration of contaminants and their plant-derived metabolites in plants across the wetland per dry mass of plants, B_{shoots} is the annual above-ground biomass produced in the wetland per unit area after the system had been operating for approximately 2 years and plant communities had matured. Biomass produced during the monitoring period was assumed to be equal to that produced during one year.

$$C_{plants}(x) = C_{measured}(x) + C_{metabolized}(x)$$

S.9

$$C_{plants}(x) = \frac{C_{measured}(x)}{(1 - f_{metabolized})}$$

S.10

In order to generate a range of possible plant uptake scenarios (Table S3), low and high B_{shoots}, $f_{metabolized}$ (the fraction of the total mass of a contaminant taken up into a plant that was eventually metabolized), and C_{plants} scenarios published in the literature[6] were used and the average of these scenarios formed the basis of the estimates reported herein. Concentrations measured in meadow plants in the summer of 2019 were estimated to represent half of the annual uptake of carbamazepine because samples were taken at approximately the midpoint of the season and concentrations increase in plants over the growing season.[6]

The contribution of plant roots to sorption was likely small because the mass of plant roots added to the subsurface per year (<700 kg)[7] is much lower than the mass of sediment in the subsurface (>6 x 10^5 kg). For example:

$$m_{roots} = \mathrm{BCF}_{root} B_{roots}\, \mathrm{K_D} w \sum \overline{C_{pw}}\, \Delta x \qquad S.11$$

$$m_{sorbed} + m_{roots} = (BCF_{root} B_{roots} + \rho_b A)\, \mathrm{K_D} \sum \overline{C_{pw}}\, \Delta x \qquad S.12$$

$$BCF_{root} B_{roots} \ll \rho_b A \qquad S.13$$

$$m_{sorbed} + m_{roots} \approx m_{sorbed} \qquad S.14$$

Where BCF_{root} is the bioconcentration factor in roots given by the ratio of the concentration of a trace organic contaminant in roots to the concentration in soil per gram of dry soil and B_{roots} is the annual below-ground biomass produced in the wetland per unit area. BCF_{root} values are much less than 10^2 for hydrophilic compounds such as those monitored in this study.[8]

S-2.1.1 Plant Uptake Scenarios

Table S3 Plant uptake scenarios used to estimate the contribution of plant uptake and translocation to the removal of trace organic contaminants in the horizontal levee.

Variable	Values tested
$\overline{C_{measured}}$[6]	Average of plant species with highest concentrations were representative of concentrations across the entire slope (e.g. concentration were uniformly high)<br><br>Average of plant species with lowest concentrations were representative across the entire slope (e.g. concentrations were uniformly low)<br><br>Average of concentrations at each location was representative of concentrations at that location. Concentrations were integrated across the slope.
B_{shoots}[7]	Mean - 1 standard deviation estimate of biomass production<br><br>Mean estimate of biomass production<br><br>Mean + 1 standard deviation estimate of biomass production
$f_{metabolized}$[9,10]	33% of the carbamazepine taken up into plants is metabolized<br><br>75% of the carbamazepine taken up into plants is metabolized
Plant species	B_{shoots} and $\overline{C_{measured}}$ estimates were made on the basis of concentrations measured in willow and meadow vegetation.

S-2.2 Estimates of HRT

In January of 2020, one of the wetland cells was converted to operation with RO concentrate. During the transition to RO concentrate, Cl^- concentrations were monitored in the effluent of the RO concentrate cell periodically (Figure S2) . Monitoring of the increase in Cl^- concentrations in the effluent of the RO concentrate cell allowed us to validate the hydraulic retention time estimates made using lithium bromide tracer injections into smaller portions of the wetland previously.[11] Using the following set of equations, we estimated that the hydraulic retention time is approximately 19 days in the RO concentrate cell. This is within our prior estimates of HRT of about 12-20 days.[11]

$$\frac{[Cl]}{[Cl]_{o_{relative}}} = \frac{[Cl]}{[Cl]_o} - \overline{\frac{[Cl]}{[Cl]_{o_{final}}}} \tag{S.15}$$

$$HRT = \sum \Delta \frac{[Cl]}{[Cl]_{o_{relative}}} * t_{avg} \tag{S.16}$$

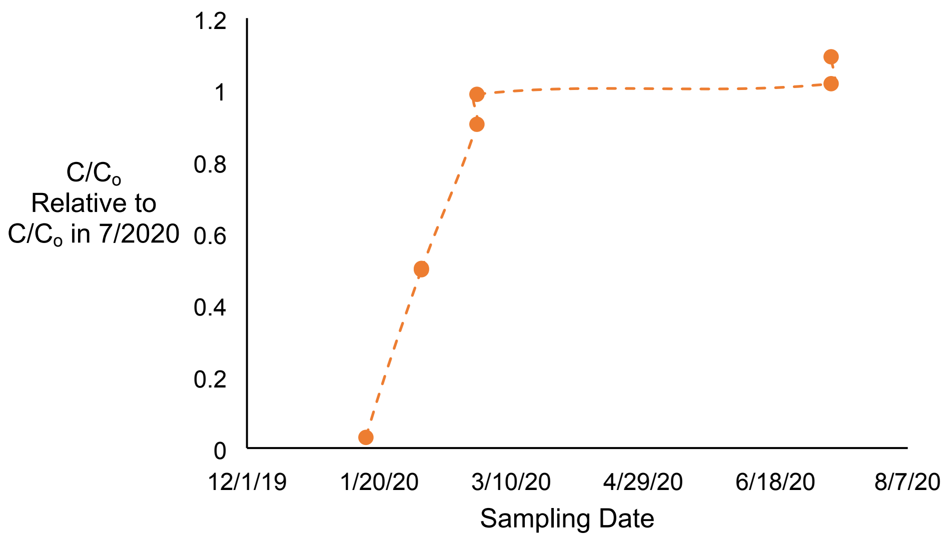

Figure S2 Increases in the relative concentration of chloride during the transition from wastewater effluent to RO concentrate. The transition to RO concentrate began on January 15, 2020. Full breakthrough was first observed on February 26, 2020.

S-2.3 Contaminant Half-Lives

To calculate contaminant half-lives, we fit first-order removal rate models to porewater data. Examples of rate fitting can be found in Figure S4 & Figure S5. This analysis assumes that the system is operated after contaminant breakthrough and, therefore sorption does not contribute to contaminant removal. In other words, contaminants loaded into each section of the wetland are removed via biotransformation (e.g., the contaminant removal profiles are no longer migrating across the wetland as they would if sorption was the primary mechanism of removal). A lack of seasonal removal and the low contribution of sorption to the mass balance suggests that the contaminant removal profiles were relatively stable in 2019 when rate constants were derived (Section S-4.2 of the SI).

S-2.4 Methods Used to Calculate Distribution Coefficients

All isotherms were linear apart from sulfamethoxazole (Figure S3). Therefore, distribution coefficients were used to estimate sorption given as the ratio between the concentration of contaminants on soil (C_s µg/g-dry soil) to the concentration in the aqueous phase at equilibrium (C_{aq} µg/L).

$$\mathrm{K_D} = \frac{C_s}{C_{aq}} \tag{S.17}$$

Isotherm K_D's were derived by measuring the mass added into each microcosm, m_{added}, and the mass measured in the aqueous phase, m_{aq}, after equilibration. The amount sorbed to the solid, m_{sorb}, was calculated as the difference between the mass added and the mass measured after equilibration.

$$m_{added} = m_{sorb} + m_{aq} \tag{S.18}$$

$$m_{added} = C_{aq}V_{aq} + C_s m_s \tag{S.19}$$

$$C_s = \frac{m_{added} - C_{aq}V_{aq}}{m_s} \tag{S.20}$$

Field- and microcosm-derived K_D's were calculated by measuring concentrations in porewater and on sediments through solid-liquid extractions. The mass of contaminants in the porewater of wet sediments was accounted for by assuming porewater concentrations were uniform in sediments using the following equation

$$C_s = C_{extract}V_{extract} - C_{aq}f_w m_{wet\ soil}\rho_w \tag{S.21}$$

Where f_w is the moisture content of the wet soil.

S-2.5 One-Dimensional Fixed Bed Sorption Modeling and Breakthrough Times

The following equation[12] describes the fate and transport of contaminants undergoing sorption in a fixed-bed column assuming empty bed conditions. Using a numerical solution to this equation, we modeled the anticipated breakthrough curve in the horizontal levee if sorption alone controlled the fate of trace organic contaminants and assumed that sorption is instantaneous (e.g., ignoring sorption kinetics). This assumption provides a conservative estimate of breakthrough time because if sorption kinetics are slower than anticipated, we would expect trace organic contaminants to exit the wetland prior to their sorption breakthrough times.

$$\frac{\partial c}{\partial t} = -\frac{v}{\phi + \rho_b \left(\frac{\partial C_s}{\partial C_{aq}}\right)_{eq}} \frac{\partial c}{\partial z}$$

S.22[12]

$$v = \frac{L_{wetland}}{HRT_{av}}$$

S.23

$$\left(\frac{\partial C_s}{\partial C_{aq}}\right)_{eq} = \mathrm{K_D}$$

S.24

Where $L_{wetland}$ and HRT_{av} are the wetland length and hydraulic retention time respectively.

The accuracy of the results of the numerical solution were validated by comparing the time at which 50% of the influent concentration was expected to appear at the outlet (e.g. breakthrough times, $t_{50\%\ breakthrough}$) to those generated by calculating retardation factors, *R*.

$$t_{50\%\ breakthrough} = R * HRT_{avg}$$

S.25

$$R = 1 + \left(\frac{\rho_b}{\phi}\right)\mathrm{K_D}$$

S.26

S-3 Redox Zonation

Redox zones were delineated in individual wetland cells to determine the fraction of contaminant removal occurring in each zone. The nitrate and oxygen reducing zone was defined as the distance from the start of the wetland to the first porewater sample where concentrations of nitrate declined to below 1mg-N/L. We defined the start of the sulfate reducing zone as the first porewater sample that had sulfate concentrations more than 10% less than the influent value and continued until concentrations declined to below 1mg/L. Contaminant removal in each zone relative to the total amount removed was calculated. Wetland cells with overlapping nitrate and sulfate reducing zones were excluded from the analysis because we could not isolate the redox communities in these sections of the wetland.

S-4 Supplementary Results

S-4.1 Sorption Isotherm Results

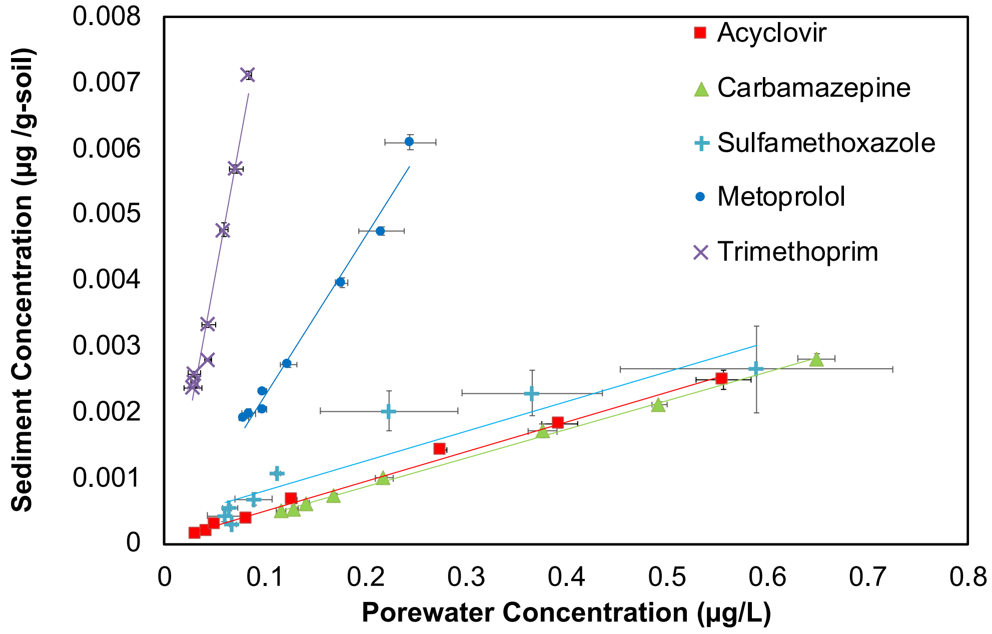

Figure S3 Isotherm results for the monitored trace organic contaminants.

Table S4 Summary of K_D values derived from isotherms, microcosms, and field results.

Compound	Field K_D	Nitrate-reducing Microcosm K_D	Sulfate-reducing Microcosm K_D	Surface Layer Isotherm-derived K_D	Treatment Layer Isotherm-derived K_D
Acyclovir	0.0046 +/- 0.0005	0.0007 +/- 0.0006	0.0015 +/- 0.0012	0.0048 +/- 0.0010	0.0040 +/- 0.0016
Carbamazepine	0.0093 +/- 0.0028	0.0025 +/- 0.0014	0.0026 +/- 0.0016	0.0032 +/- 0.0005	0.0052 +/- 0.0050
Carboxy-acyclovir	0.0011 +/- 0.0012	0.0005 +/- 0.0003	0.0004 +/- 0.0001	0.0027 +/- 0.0003	0.0018 +/- 0.0234
Sulfamethoxazole	0.0026 +/- 0.0020	0.0002 +/- 0.0003	0.0017 +/- 0.0021	0.0012 +/- 0.0002	0.0043 +/- 0.0035
Metoprolol	0.0034 +/- 0.0013	0.0017 +/- 0.0006	0.0127 +/- 0.0052	0.0150 +/- 0.0034	0.0212 +/- 0.0042
Carboxy-metoprolol	0.0012 +/- 0.0005	0.0006 +/- 0.0002	0.0005 +/- 0.0002	Negligible sorption	Negligible sorption
Trimethoprim	0.0051 +/- 0.0035	0.0047 +/- 0.0020	0.0315 +/- 0.0262	0.0376 +/- 0.019	0.0259 +/- 0.0152
10-OH Carbamazepine	n.d.	0.0003 +/- 0.0004	0.0007 +/- 0.0004	n.d.	n.d.
DiOH-Carbamzepine	n.d.	0.0006 +/- 0.0005	0.0006 +/- 0.0004	n.d.	n.d.

S-4.2 Impact of Wetland Cell Type and Temperature on Performance

Because the horizontal levee removed trace organic contaminants via biotransformation, water temperature and wetland cell design (e.g., hydraulic residence time) could be important to system performance. For example, the temperature of the wetland porewater varied by about 7 degrees between winter and summer during the monitoring period (i.e., from approximately 9 to 16˚C). Microbial activity typically decreases by about 40% over this temperature change[13] though this estimate may vary significantly between sediment microbial communities.[14]

Porewater concentrations from the summer of 2019 and winter of 2019 showed that all trace organic contaminants were removed at similar rates in the summer and winter (Figure S4). Seasonal shifts in biogeochemistry may have contributed to enhanced winter removal of trace organic contaminants because sulfate-reducing conditions were important for their transformation. For example, during winter, the zone in which nitrate was reduced was not significantly different than the zone in the summer.[15] As the activity of heterotrophic organisms declined, autotrophic nitrate-reducing organisms that employ sulfide minerals as an energy source contributed more to nitrate reduction in winter. The increased activity of microorganisms performing sulfide-driven denitrification also supplied additional sulfate to the subsurface. This additional supply of sulfate may have allowed for more sulfate-reducing activity in the winter month.

A lack of seasonal contaminant removal trends suggests that other seasonal factors such as plant senescence or changes in rhizosphere-associated microbial communities were not important for the removal of trace organic contaminants. However, there is a need to study subsurface systems like horizontal levees under conditions that exhibit greater seasonal variations in temperature and microbial activity.

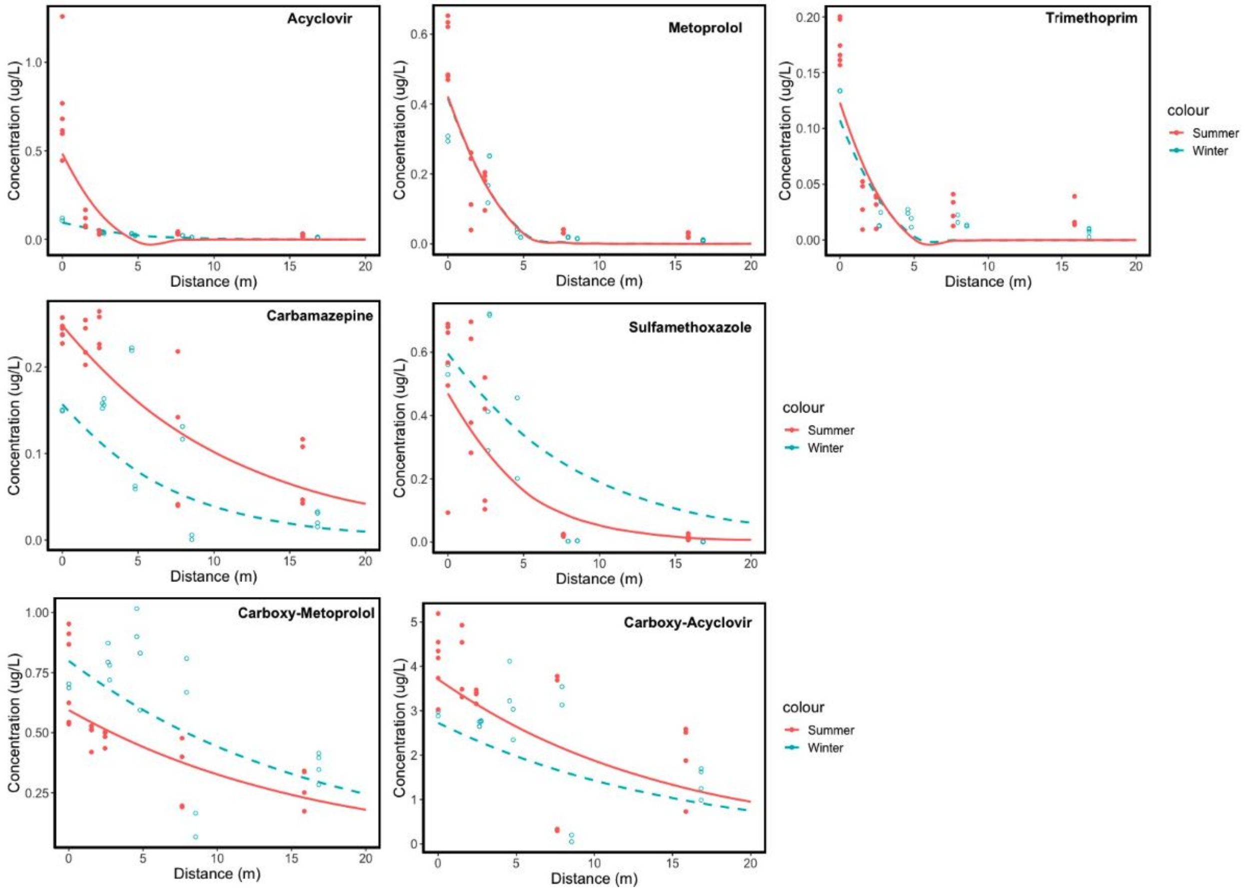

Figure S4 Concentrations of trace organic contaminants versus distance in horizontal levee inflow and throughout wetland cells. Seasonal data were collected from fine cells in winter and summer of 2019. Rate constants did not display statistically significant differences in winter and summer, except for acyclovir, but this is due to low concentrations in the inflow during the winter of 2019.

Different design parameters in each wetland cell type may have influenced horizontal levee performance by changing subsurface mass loading and hydraulic retention times. For example, despite having the same hydraulic grade line, willow cells with coarse sediments exhibited higher subsurface flows (3.6 m^3/d) than coarse meadow (2.3 m^3/d) or fine meadow (1.4 m^3/d) cells during the period when flows were adjusted so that overland flow was eliminated from the wetland cells.[11] Monitoring of concentrations of trace organic contaminants at the outlet of the wetland cells from the second year of operation showed that the percentage removal of trace organic contaminants in each wetland cell type is similar (Figure 2). However, because loading into each cell was different, the willow and coarse cells removed a significantly higher mass of contaminants than fine cells.

In addition, porewater samples revealed modest differences among cell types. For example, compounds that were removed under sulfate reducing conditions (e.g., carbamazepine) required longer distances for removal in wetland cell types that had sulfate reduction over larger areas (e.g., willow cells) due to higher flow rates and presumably shorter hydraulic retention times in those cells. In contrast, compounds that were removed under nitrate reducing conditions (e.g. metoprolol) were removed over approximately the same distance in all wetland cell types (Figure S5).

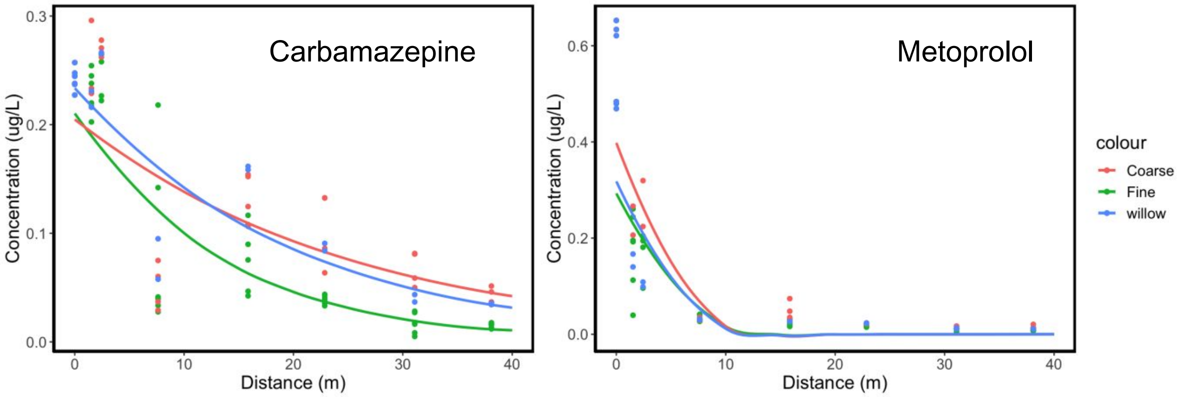

Figure S5 Concentrations of carbamazepine and metoprolol versus distance in horizontal levee influent and throughout wetland cells. Data from all three wetland cell types collected in the Summer of 2019 were plotted along with pseudo first order rate fits. Carbamazepine removal rate constants were statistically significantly different, but the magnitude was modest. Metoprolol removal rate constants were not significantly different.

S-4.3 Mass Balance Results

Analysis of data from sorption isotherm experiments (Figure S3) suggested that trace organic contaminant removal could not be explained by partitioning to sediments. For example, assuming that sediments were at equilibrium with measured porewater concentrations, predictions made with isotherm data from the treatment layer (i.e., K_D values, Table S4) indicated that sorption accounted for less than 12% of the mass of all compounds that entered the wetland between August 2018 and July 2019 other than trimethoprim and metoprolol (Figure S6). For metoprolol and trimethoprim, less then 45% of the inflow mass could have been removed by sorption. (SI S-2.1 for calculations) However, this analysis only serves to demonstrate that sorption cannot explain the observed removal, not as a quantification of sorption that did occur during the monitoring period. This approach inherently overestimates the contribution of sorption during the monitoring period because contaminant concentrations were relatively stable in wetland porewater during this period.

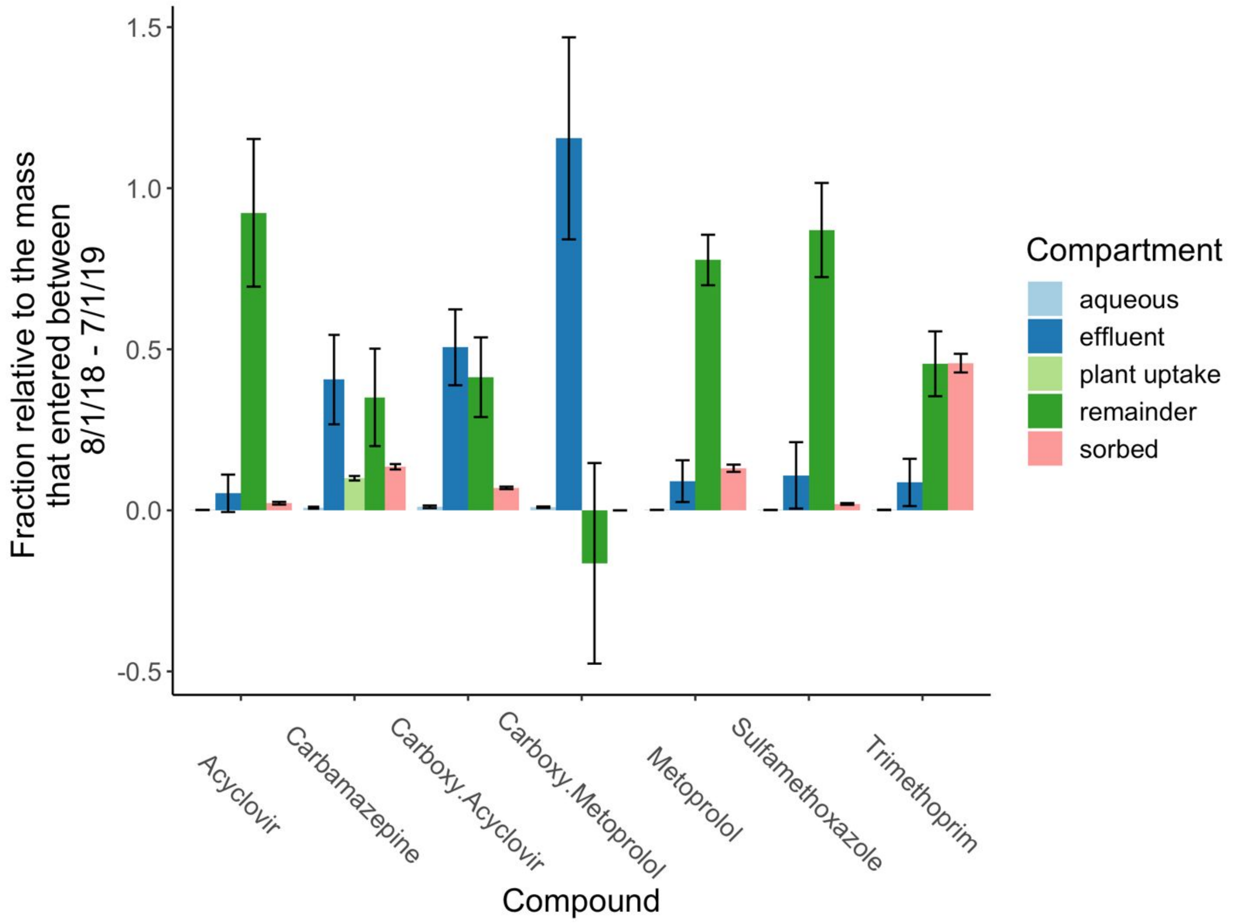

Figure S6 Mass of monitored trace organic contaminants as a fraction of that which entered the wetland between August of 2018 and July of 2019. The mass fraction in porewater, effluent, plant uptake and sorption wetland compartments were determined based on measurements whereas removal via transformation was estimated by subtraction.

To predict contaminant breakthrough, estimates of hydraulic retention time made with a lithium bromide tracer[11] were validated over the full length of the wetland by monitoring chloride concentrations when the water source of one wetland cell was converted to reverse osmosis (RO) concentrate generated from municipal wastewater reuse (Figure S2). Using this information along with sorption data, we predicted that breakthrough (i.e., the occurrence of effluent contaminant concentrations that were at least 50% of the inflow concentrations) should have occurred for sulfamethoxazole, acyclovir, and carbamazepine within 8 to 12 months of introducing municipal wastewater effluent into the subsurface wetland, which was 4-8 months prior to the date when the first samples were collected. The two compounds with the highest affinities for the sediments in the sand and gravel layer, trimethoprim and metoprolol, were estimated to have travelled approximately 6 to 9 meters and 20 to 30 meters, respectively, by the time that the porewater samples were collected if sorption controlled their removal. Although complete breakthrough was not expected for these compounds, we conclude that sorption was only of modest significance for metoprolol and trimethoprim because their concentrations decreased by over 90% within the first 5 meters of the wetland cells. (Figure 3 See SI S-2.5 for calculations)

Additional evidence for the limited role of sorption was obtained by measuring trace organic contaminants in sediments collected approximately 5 meters and 20 meters from the inlet of the wetland in March 2021 (i.e., 47 months after the system started receiving wastewater effluent) and in microcosm experiments. Field-derived and microcosm-derived K_D values were either similar to or considerably lower than isotherm-derived K_D values, suggesting that the mass balance estimates based upon laboratory isotherms provided a conservative estimate of the contribution of sorption to the removal of trace organic contaminants (Table S4).

Analysis of concentrations of trace organic contaminants in wetland vegetation indicated that plant uptake, translocation and in-planta transformation also did not account for a significant fraction of the observed removal of trace organic contaminants. Among the compounds present in the wetland porewater, those that are uncharged and of intermediate hydrophobicity (i.e., log K_{ow} ranging from 2 to 4) are most susceptible to plant uptake and translocation via the xylem.[16,17] Due to their physicochemical characteristics, only a small fraction of the trace organic contaminants present in the porewater entered the plants because they were partially or completely excluded from the transpiration stream due to difficulty bypassing the casparian strip to enter the xylem. Among the contaminants monitored in this study, carbamazepine was expected to have the highest levels of uptake due to its physicochemical characteristics (Table S2) and persistence in the horizontal levee porewater (Figure 3). Also, carbamazepine and its metabolites were the only monitored contaminants that were consistently detected in wetland plant samples.[6] Therefore, we assumed that plant uptake and translocation was negligible for all contaminants apart from carbamazepine.

Using carbamazepine plant concentrations measured in various plant species reported elsewhere,[6] estimates of in-planta metabolism[9,10] and biomass measurements,[7] we estimated that plant uptake and translocation removed less than 12% of the carbamazepine that entered the horizontal levee subsurface (Figure S6, SI S-2.1 for calculations). This estimate is similar to estimates of plant uptake and translocation of carbamazepine made in other soil-plant systems.[18]

S-4.4 Microcosm Experiments

S-4.4.1 Microcosm Methods

Microcosm experiments were performed using fresh sediments collected from the subsurface treatment layer. Sediments from the treatment zone of the nitrate-reducing region and the sulfate-reducing region were placed in plastic containers without headspace, stored on ice and transported back to the lab. Porewater that would later be spiked with trace organic contaminants and terminal electron acceptors for use in the microcosms was collected from approximately 3 meters from the outlet of the wetland. Porewater was collected at each sampling location and paired with sediment extractions prior to the microcosm experiments to determine the initial background mass of trace organic contaminants in the microcosms.

In the lab, approximately 30 g of well-homogenized sediments of each type were subsampled into pre-weighed 20-mL glass scintillation vials, after removing gravel larger than 0.5 cm from the sediment. Approximately 2 mL of a spiked-wetland porewater solution containing

the corresponding terminal electron acceptor and a mixture of the trace organic contaminants (approximately 50 µg/L of each) without any residual solvent was added to each vial. Experimental treatments are outlined in Table S5. The time between sediment collection and initiation of the experiment was approximately 10 hours.

Microcosms were incubated in the dark at room temperature (23ºC +/- 0.7ºC). Porewater was collected in triplicate by sacrificially sampling microcosms. Incubations lasted 2 days in the nitrate-reducing microcosms and over 28 days in the sulfate-reducing microcosms. Trace organic contaminants were extracted from microcosm sediments collected before and during incubation. A detailed sampling and experimental plan for the microcosm experiments are presented in Table S6.

Table S5 Experimental treatments and corresponding shorthand used for labels in Table S6

Treatments	Shorthand	#of time points	Total Vials needed
Nitrate + nitrate reducing soil + spiked porewater	N#	6	18
Sulfate + sulfate reducing soil + spiked porewater	S#	12	36
Control - no soil + nitrate + spiked porewater	NS-N#	3	9
Control - no soil + sulfate + spiked porewater	NS-S#	3	9
Control - nitrate reducing soil + spiked porewater	NN#	3	9
Control - sulfate reducing soil + spiked porewater	NS#	3	9

Table S6 Sampling plan for the microcosm experiments including plans for periodic extraction and porewater sampling

Time point	Hour #	Days	Weeks	Day of week/time target	Description	Samples taken (description)	Porewater	Soil Extract	N	NS-N	NN	S	NS-S	NS
t0	0	0.0	0.0	Wednesday March 17	sample before introducing anything into the microcosms (soil alone, porewater alone)	unamended porewater, porewater + nitrate, porewater + sulfate, porewater + sulfate+CBZ, triplicate unamended soil extracts, triplicate unamended soil microbial	4	3						
t1	1	0.0	0.0	Wednesday March 17	sorption test	porewater, soil	6	6	pw+s			pw+s		
t2	3	0.1	0.0	wednesday March 17		poerwater	6		pw			pw		
t3	17	0.7	0.1	Thursday, March 18 AM	peak nitrate reducing	porewater, soil	14	11	pw+s	pw	pw+s	pw+s		
t4	24	1.0	0.1	Thursday, March 18 PM	peak nitrate reducing	poerwater	9		pw	pw	pw			
t5	41	1.7	0.2	Friday March 19, AM	near end nitrate reducing	porewater, soil	14	11	pw+s			pw+s	pw	pw+s
t6	47	2.0	0.3	Friday March 19, PM	end nitrate reducing	poerwater	9		pw	pw	pw			
t7	113	4.7	0.7	Monday March 22 AM	s reducing	poerwater	5					pw		
t8	185	7.7	1.1	Thursday March 25 AM	s reducing	poerwater	3					pw		
t9	281	11.7	1.7	Monday March 29 AM	s reducing	poerwater	3					pw		
t10	353	14.7	2.1	Thursday April 1 AM	peak S reducing	porewater, soil	11	8				pw+s	pw	pw+s
t11	449	18.7	2.7	Monday april 5 AM	s reducing	poerwater	3					pw		
t12	521	21.7	3.1	Thursday april 8 am	s reducing	poerwater	3					pw		
t13	617	25.7	3.7	Monday april 12 AM	s reducing	poerwater	3					pw		
t14	689	28.7	4.1	Thursday april 15 AM	end S reducing	porewater, soil	11	8				pw+s	pw	pw+s
						sum -->	104	47						

S-4.4.2 Microcosm Results

We used microcosm experiments to test the removal of trace organic contaminants under different redox conditions because some compounds (e.g., acyclovir, 10-OH carbamazepine and trimethoprim) were fully removed before they reached the sulfate-reducing zone in the full-scale wetland (Figure 3). Analysis of the total mass of trace organic contaminants in microcosm porewater and soil extracts allowed us to attribute removal in the microcosms to transformation.

Acyclovir, trimethoprim, and 10-OH carbamazepine, three compounds that were depleted within the first five meters of the inlet in the wetland, were removed in microcosms that contained sediment from both the nitrate-reducing zone and the sulfate-reducing zone (Figure S8a, Figure S9a). These results are consistent with previous reports in which acyclovir was removed under anaerobic conditions in membrane bioreactors[19] and trimethoprim was removed under anoxic and anaerobic conditions in lab-scale experiments.[20] However, 9-acridine carboxylic acid, a metabolite of 10-OH carbamazepine, increased and was quickly removed in only the microcosms containing sediment from the nitrate-reducing zone suggesting that the 10-OH carbamazepine degradation pathway differed under nitrate-reducing and sulfate-reducing conditions in the microcosms.

The removal of trace organic contaminants in the microcosms containing sediments collected from the nitrate-reducing zone was more modest than the removal observed in the field likely because only 2.5 mg-N/L of nitrate was available to nitrate-reducing microbial communities in the microcosms. This concentration of nitrate was rapidly removed within 20 hours (i.e.. the first trace organic contaminant sampling point in Figure S8a) indicating that nitrate-reducing conditions were not predominant for the period between 20 and 48 hours.

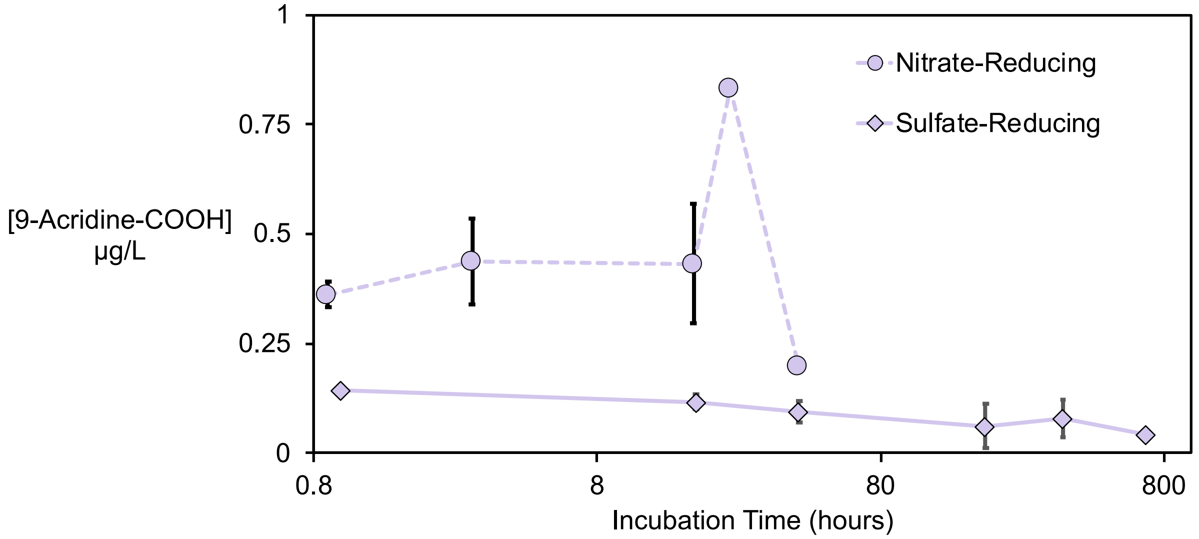

Figure S7 9-Acridine-COOH was formed in the microcosms that contained sediment from the nitrate-reducing zone, but not in the microcosms that contained sediment from the sulfate-reducing zone.

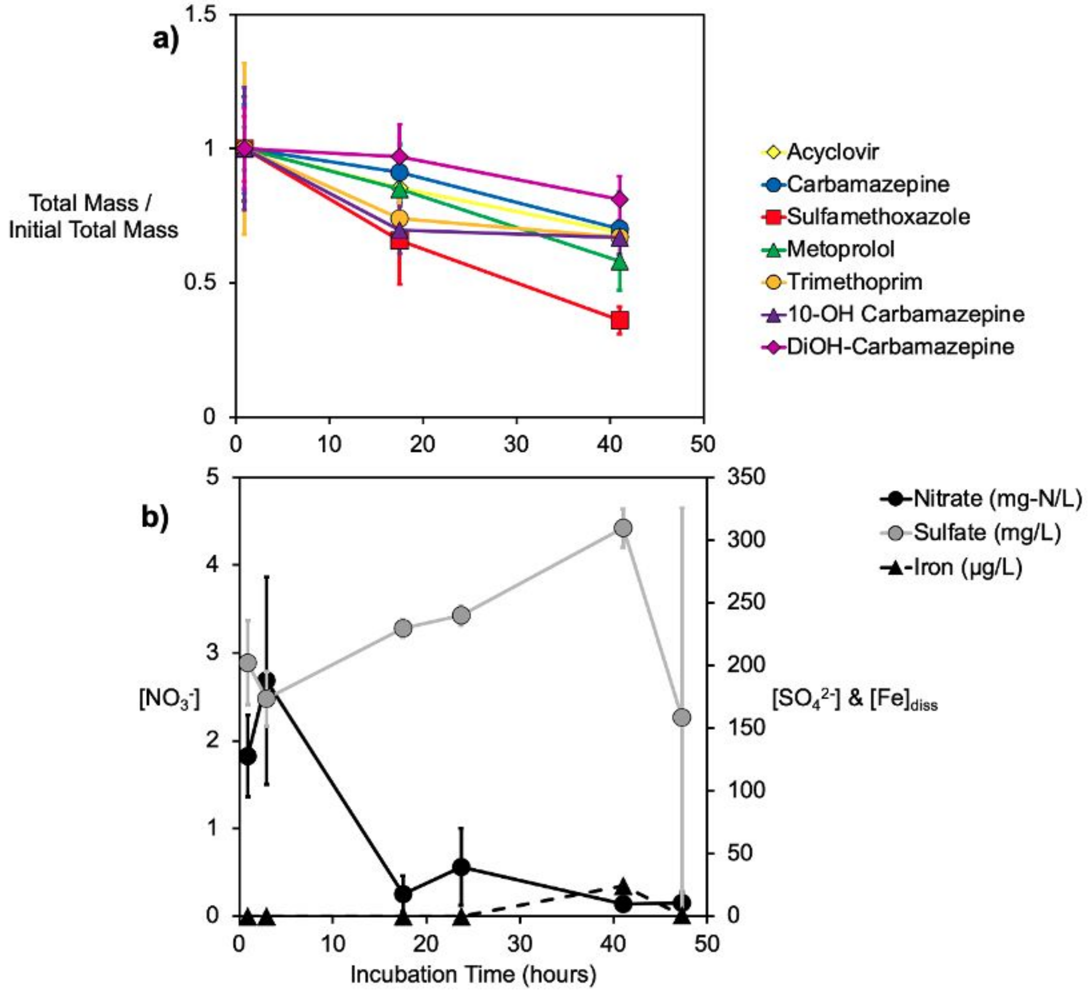

Figure S8 Total mass of trace organic contaminants remaining relative to initial total mass after the first sampling point in microcosms containing sediment from the nitrate-reducing zone (a) and concentrations of redox-active inorganic species (b). Iron concentrations are represented by the dashed symbol.

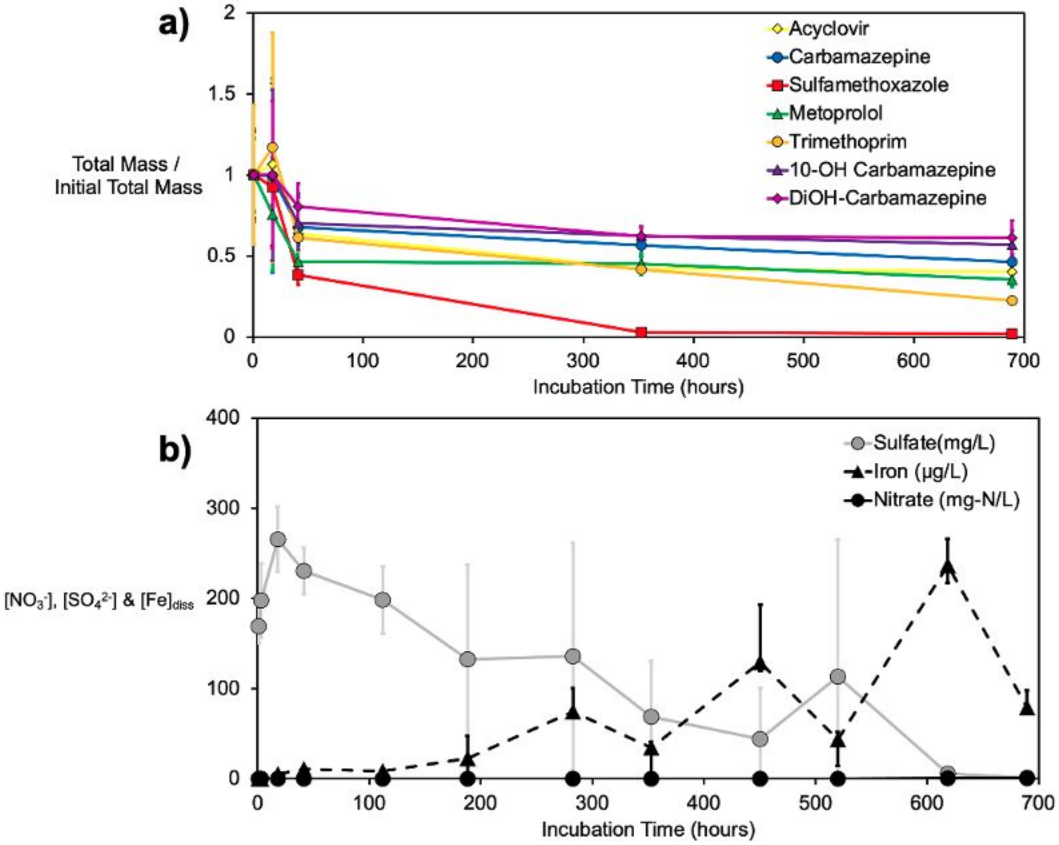

Figure S9 Total mass of trace organic contaminants relative to initial total mass after the first sampling point in microcosms containing sediment from the sulfate-reducing zone (a). Concentrations of redox indicator species are also plotted over time (b). Iron concentrations are represented by the dashed symbol.

Persistent trace organic contaminants (e.g., carbamazepine and sulfamethoxazole) were also removed in microcosms that contained sediments collected from the nitrate-reducing and the sulfate-reducing zones. For sulfamethoxazole, this finding is consistent with prior research that indicates it is removed under nitrate, iron, and sulfate-reducing conditions via biotransformation and reactions with microbially-produced reactive species (e.g., adsorbed Fe(II)).[21–23] Carbamazepine was only removed in these microcosms after nitrate decreased to less than 0.5mg-N/L in the microcosms (e.g., after 20 hours). After this period, sulfate and iron reduction may have occurred in these microcosms (Figure S8b). These results were also consistent with our field results.

S-4.5 Other Supplemental Results

	Carbamazepine	10-OH Carbamazepine		Dihydroxy Carbamazepine
	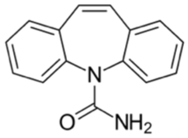	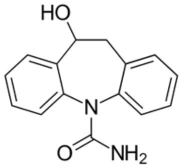		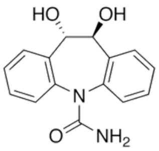
Aerobic Conditions (0-1.5m in horizontal levee)	Persistent	Removed	Formed 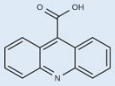 9-Acridine-COOH	Slowly Removed
Nitrate-reducing conditions (0-10m in horizontal levee)	Persistent	Removed	Formed 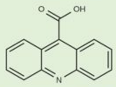 9-Acridine-COOH	Slowly Removed
Sulfate-reducing & iron-reducing conditions (5-30m in horizontal levee)	Removed	Removed*	Removed	Slowly Removed

Figure S10 Redox dependent removal of carbamazepine and related hydroxylated compounds. Hydroxylation of carbamazepine alters the rates and pathways of carbamazepine transformation under diverse redox conditions. 9-Acridine-COOH was only formed under aerobic and nitrate-reducing conditions. *only observed in microcosm experiments.

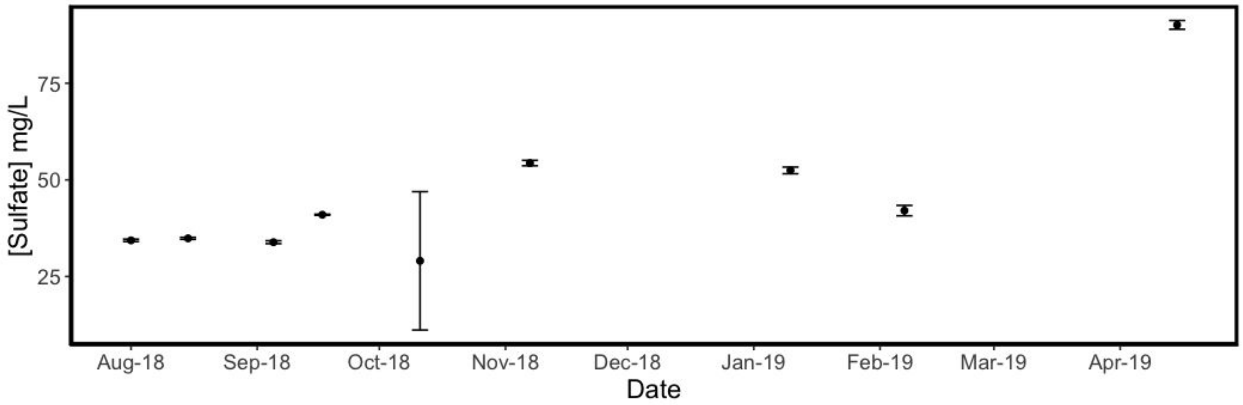

Figure S11 Inlet sulfate concentrations over the monitoring period.

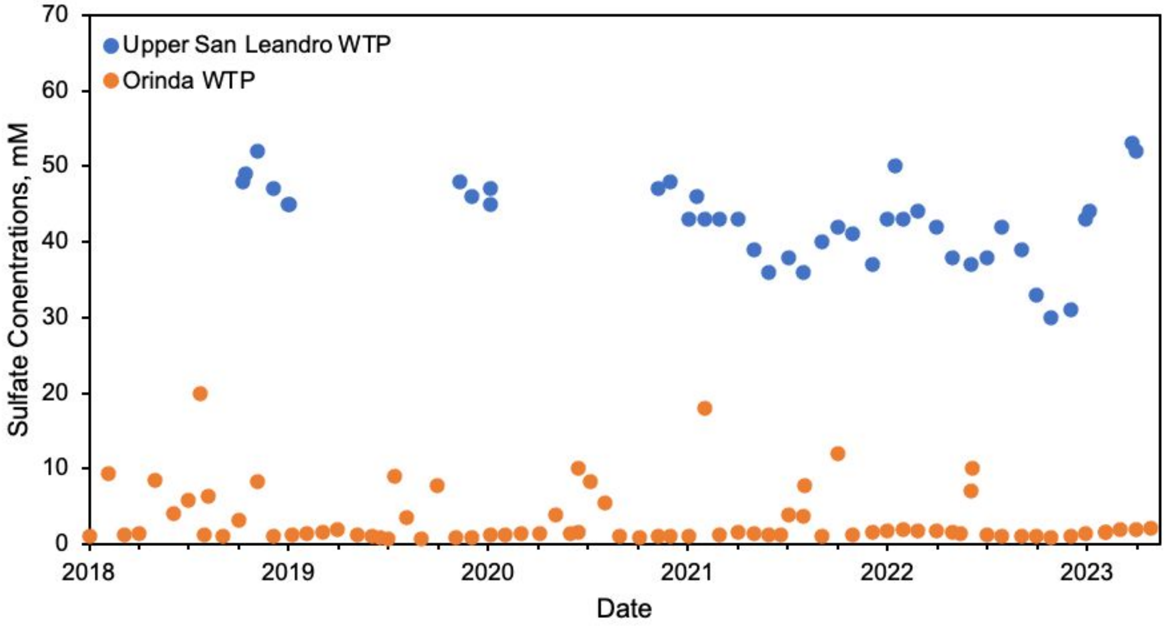

Figure S12 Sulfate concentrations measured during the monitoring period at the two water treatment plants: the Orinda WTP and the Upper San Leandro WTP, that supply drinking water to the service area that produces wastewater treated at the Oro Loma Sanitary District wastewater treatment plant. The drinking water at the two treatment facilities have significantly different concentrations of sulfate (e.g., median sulfate concentrations of 41 mg/L and 1.5 mg/L, respectively). During the monitoring period, the Upper San Leandro WTP was taken of service during periods of lower demand or to complete upgrades to the facilities. In contrast, the Orinda WTP remained in service at most times, except when taken out of service for critical maintenance. The difference in sulfate concentrations at the two drinking water treatment facilities partly explains the variation in sulfate concentrations observed in the inflow to the horizontal levee.

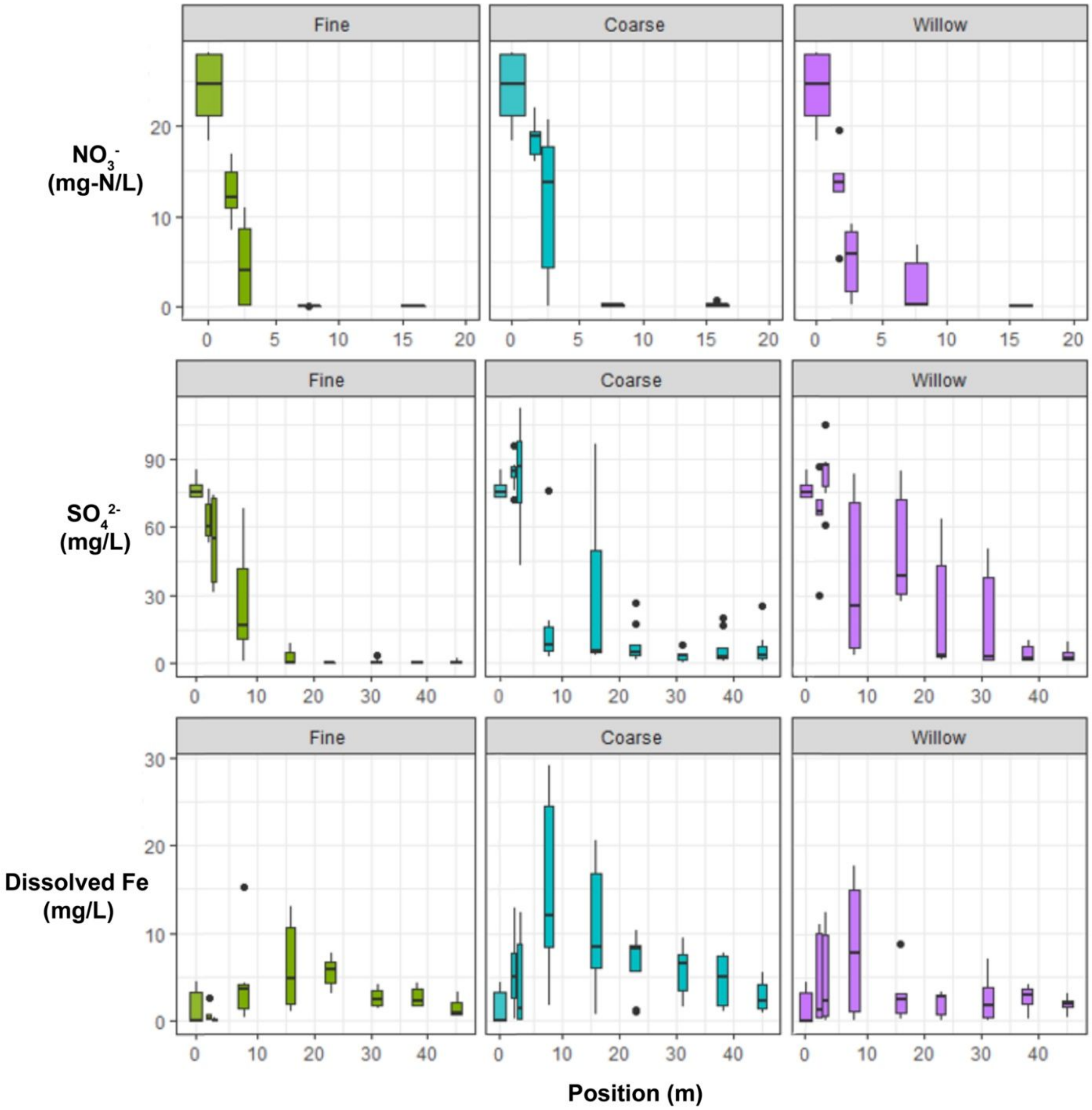

Figure S13 Redox conditions as indicated by the concentrations of nitrate, dissolved iron in field-filtered samples, and sulfate in all three wetland cell types- redrawn from Cecchetti et al.[15]

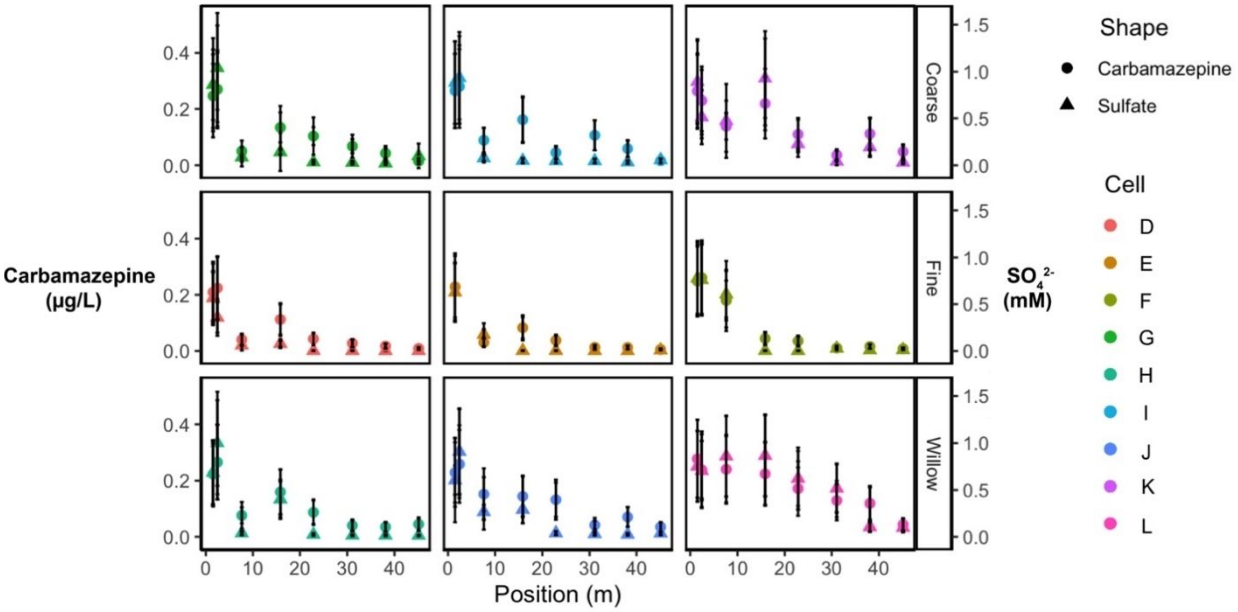

Figure S14 Mean concentrations of carbamazepine (dots) and sulfate (triangles) in the influent and coarse, fine, and willow cells along the flow path. Error bars represent the range of measured concentrations at each sampling location.

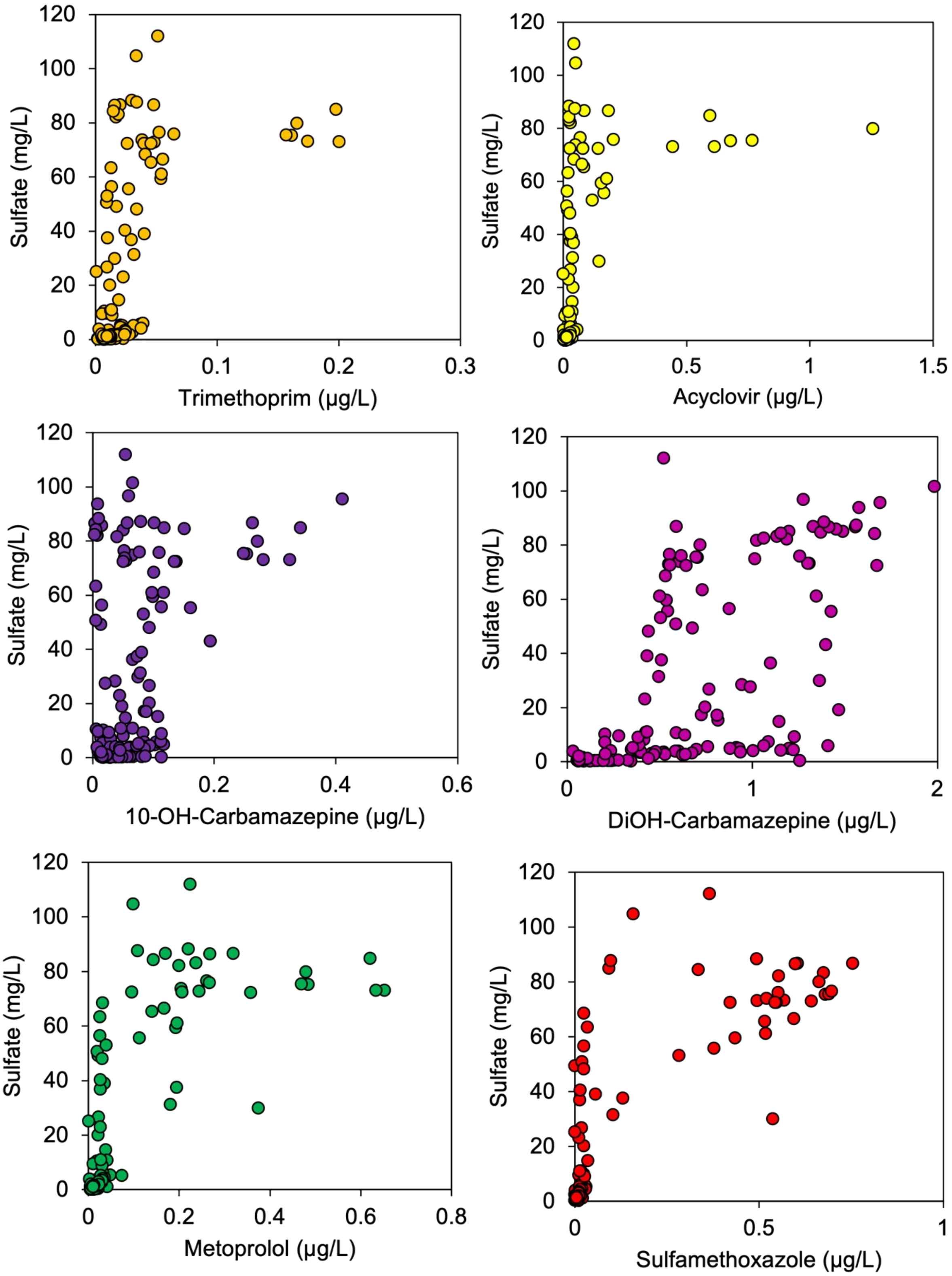

Figure S15 Scatter plots showing trace organic contaminant concentrations and sulfate

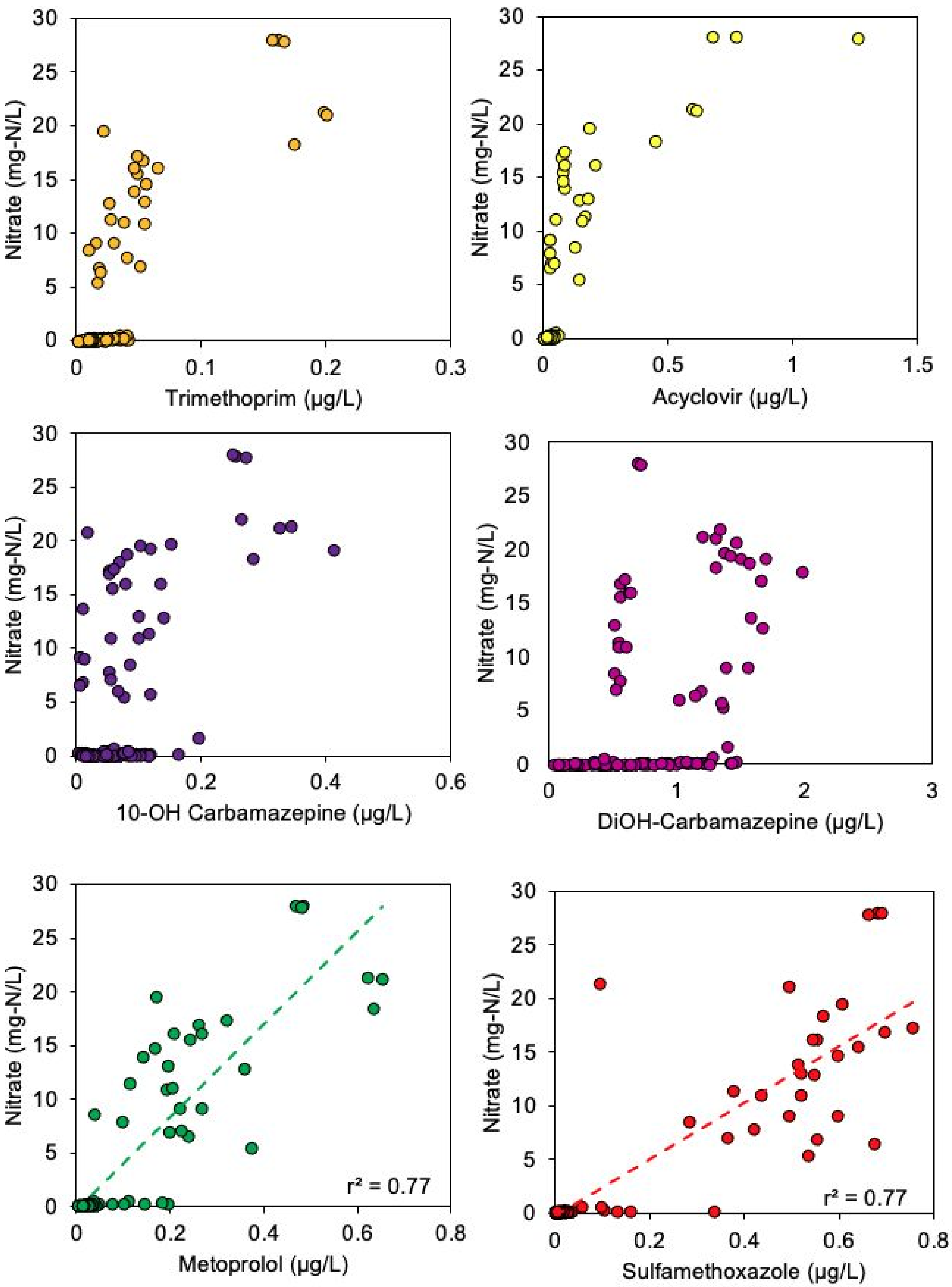

Figure S16 Scatterplots showing trace organic contaminant concentrations and nitrate

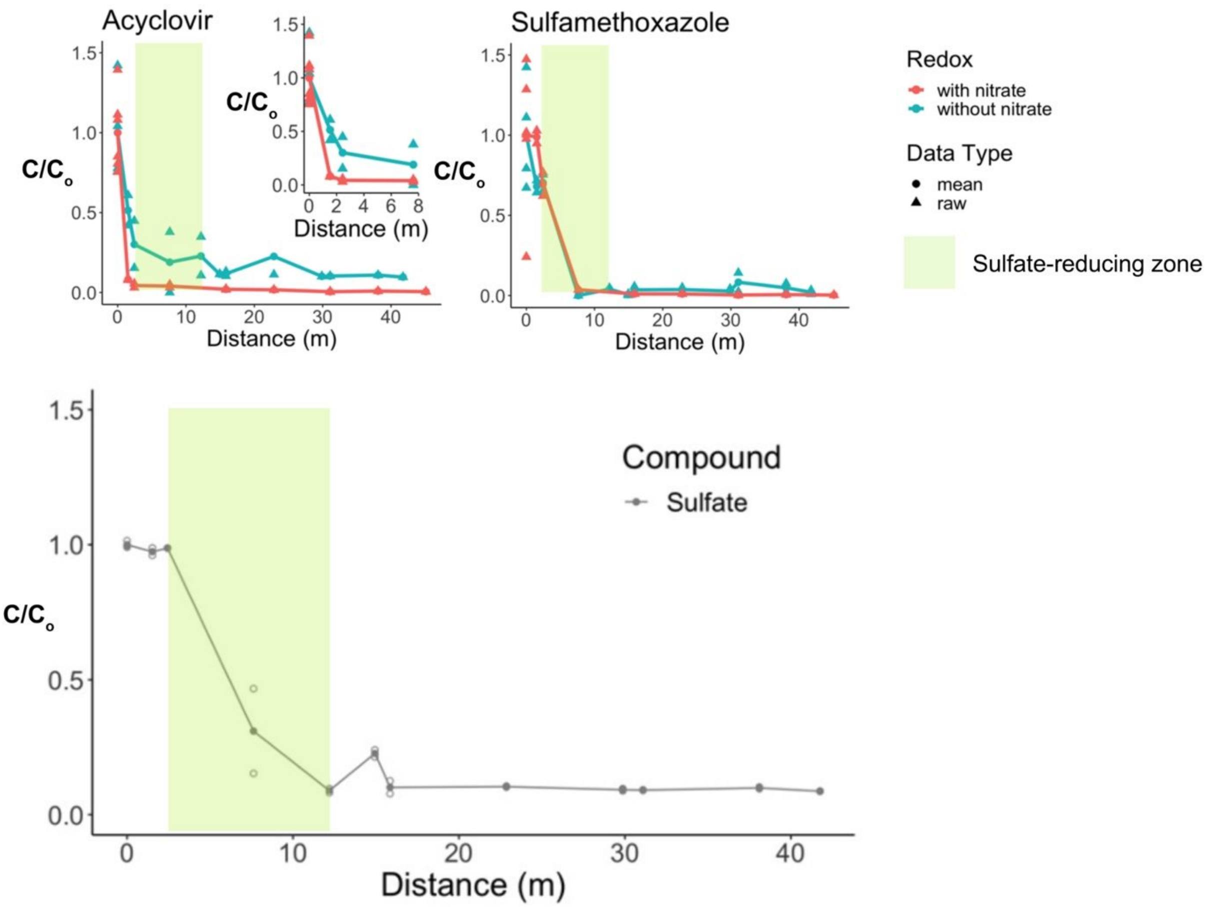

Figure S17 Results from sampling before and after treatment plant upgrades. Trimethoprim concentrations were significantly lower in the inflow to the wetland after nitrate concentrations declined, which we anticipated because trimethoprim removal is enhanced in treatment plants that employ anoxic biological nutrient removal,[24,25] but this prevented our ability to interpret the impacts of upstream treatment upgrades to its transformation from field data in the wetland.

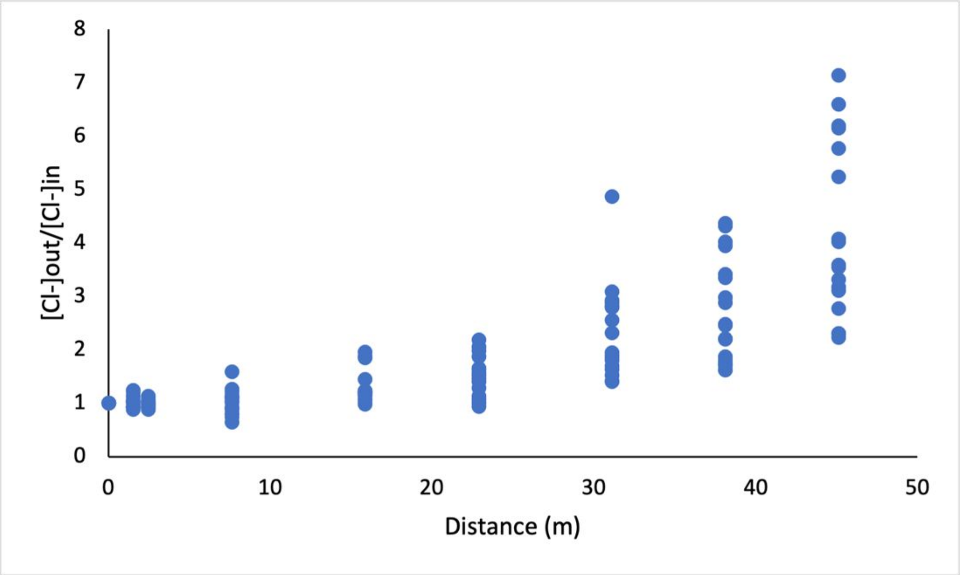

Figure S18 Ratio of chloride in the outflow to chloride in the inflow used to normalize concentrations for evapotranspiration.

S-5 Photos of the Field Site

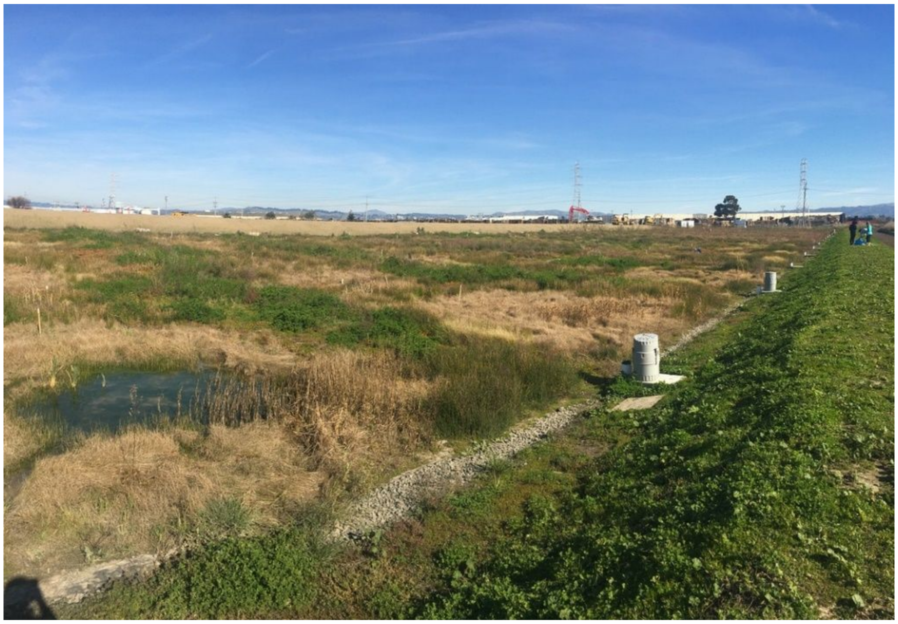

Figure S19 Horizontal levee in January 2017 view from the effluent sampling wells

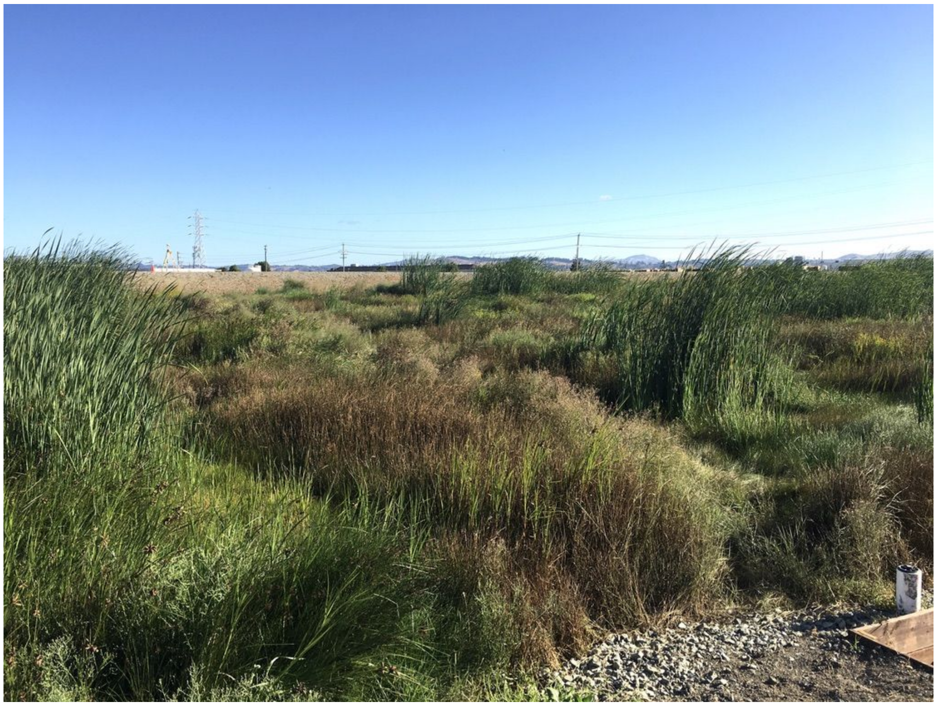

Figure S20 Horizontal levee in October 2017 - view from the effluent sampling wells

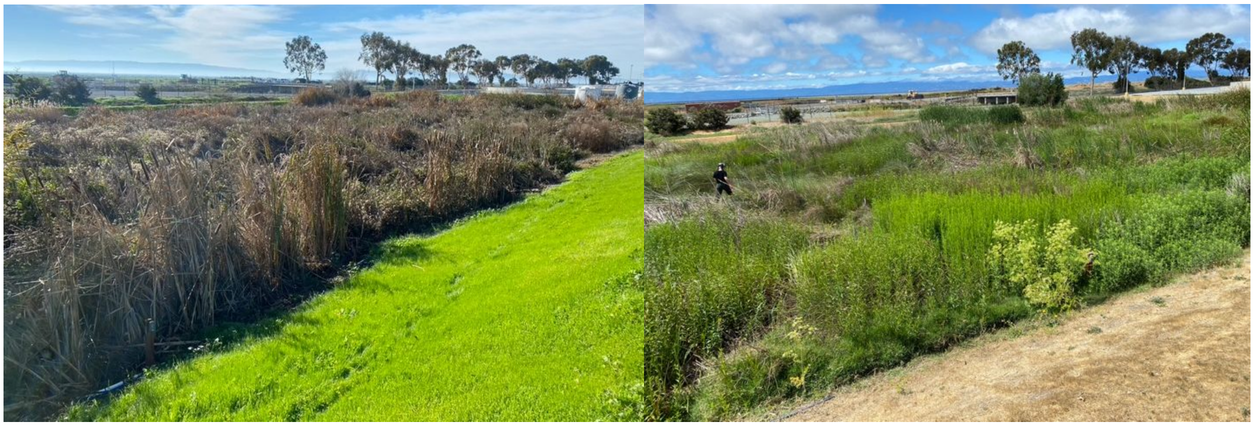

Figure S21 Horizontal levee in winter of 2022 (left) and summer of 2021 (right) at approximately the same location near the inlet

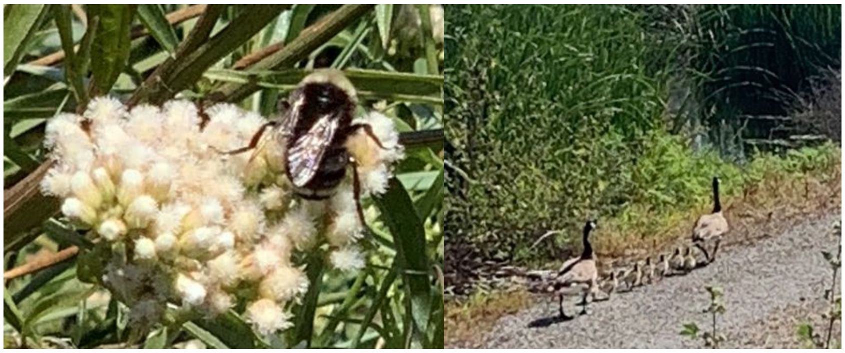

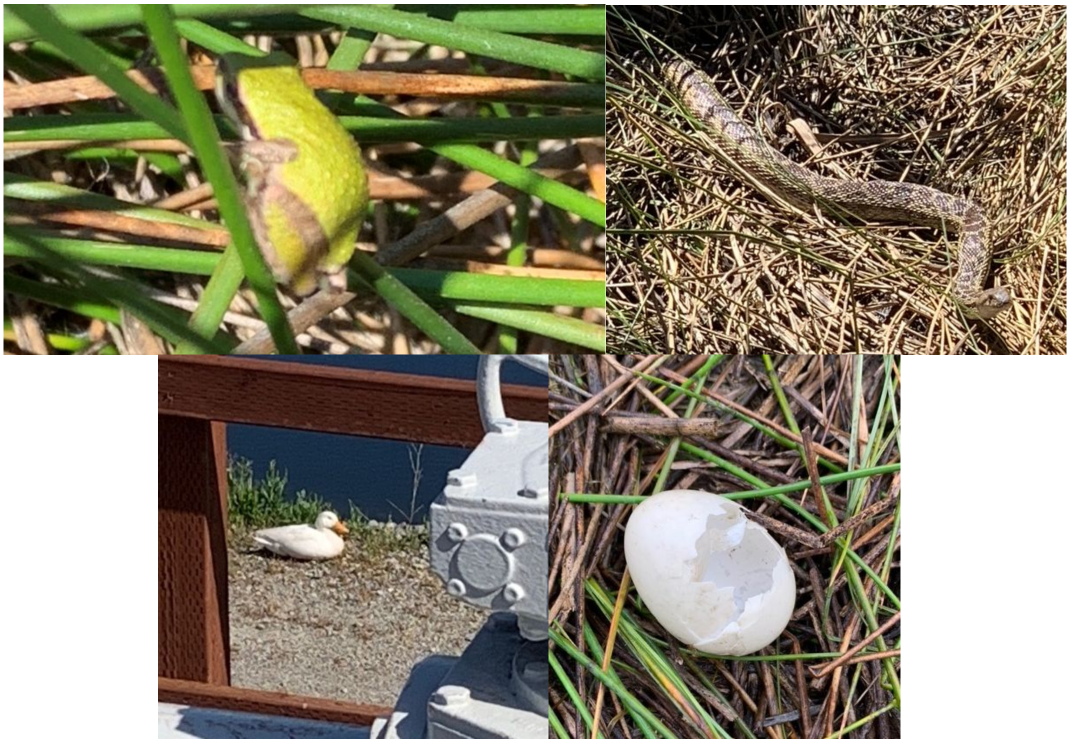

Figure S22 Wildlife use of the horizontal levee field site. A variety of other birds, insects, and mammals (e.g. jackrabbits) were also seen onsite but are not pictured.

S-6 References

(1) Thomas, D. H.; Rey, M.; Jackson, P. E. Determination of Inorganic Cations and Ammonium in Environmental Waters by Ion Chromatography with a High-Capacity Cation-Exchange Column. *J. Chromatogr. A* **2002**, *956* (1–2), 181–186. https://doi.org/10.1016/S0021-9673(02)00141-3.

(2) APHA; AWWA; WEF. *Standard Methods for Examination of Water and Wastewater. 22nd Ed. Washington: American Public Health Association*; 2012. https://doi.org/ISBN 978-087553-013-0.

(3) Prasse, C.; Wenk, J.; Jasper, J. T.; Ternes, T. A.; Sedlak, D. L. Co-Occurrence of Photochemical and Microbiological Transformation Processes in Open-Water Unit Process Wetlands. *Environ. Sci. Technol.* **2015**, *49* (24), 14136–14145. https://doi.org/10.1021/acs.est.5b03783.

(4) Bear, S. E.; Nguyen, M. T.; Jasper, J. T.; Nygren, S.; Nelson, K. L.; Sedlak, D. L. Removal of Nutrients, Trace Organic Contaminants, and Bacterial Indicator Organisms in a Demonstration-Scale Unit Process Open-Water Treatment Wetland. *Ecol. Eng.* **2017**, *109*, 76–83. https://doi.org/10.1016/j.ecoleng.2017.09.017.

(5) Jasper, J. T.; Jones, Z. L.; Sharp, J. O.; Sedlak, D. L. Biotransformation of Trace Organic Contaminants in Open-Water Unit Process Treatment Wetlands. *Environ. Sci. Technol.* **2014**, *48* (9), 5136–5144. https://doi.org/10.1021/es500351e.

(6) Stiegler, A. N.; Cecchetti, A. R.; Sedlak, D. L. Plant Uptake of Trace Organic Contaminants in Effluent-Dominated Streams: An Overlooked Terrestrial Exposure Pathway. *Environ. Sci. Technol. Lett.* **2022**, *9* (11), 929–936. https://doi.org/10.1021/acs.estlett.2c00543.

(7) Cecchetti, A. R.; Sytsma, A.; Stiegler, A. N.; Dawson, T. E.; Sedlak, D. L. Use of Stable Nitrogen Isotopes to Track Plant Uptake of Nitrogen in a Nature-Based Treatment System. *Water Res. X* **2020**, *9*, 100070. https://doi.org/10.1016/j.wroa.2020.100070.

(8) Doucette, W. J.; Shunthirasingham, C.; Dettenmaier, E. M.; Zaleski, R. T.; Fantke, P.; Arnot, J. A. A Review of Measured Bioaccumulation Data on Terrestrial Plants for Organic Chemicals: Metrics, Variability, and the Need for Standardized Measurement Protocols. *Environ. Toxicol. Chem.* **2018**, *37* (1), 21–33. https://doi.org/10.1002/etc.3992.

(9) Riemenschneider, C.; Seiwert, B.; Moeder, M.; Schwarz, D.; Reemtsma, T. Extensive Transformation of the Pharmaceutical Carbamazepine Following Uptake into Intact Tomato Plants. *Environ. Sci. Technol.* **2017**, *51* (11), 6100–6109. https://doi.org/10.1021/acs.est.6b06485.

(10) Goldstein, M.; Malchi, T.; Shenker, M.; Chefetz, B. Pharmacokinetics in Plants: Carbamazepine and Its Interactions with Lamotrigine. *Environ. Sci. Technol.* **2018**, *52* (12), 6957–6964. https://doi.org/10.1021/acs.est.8b01682.

(11) Cecchetti, A. R.; Stiegler, A. N.; Graham, K. E.; Sedlak, D. L. The Horizontal Levee: A Multi-Benefit Nature-Based Treatment System That Improves Water Quality and Protects Coastal Levees from the Effects of Sea Level Rise. *Water Res. X* **2020**, *7*, 100052. https://doi.org/10.1016/j.wroa.2020.100052.

(12) Benjamin, M.; Lawler, D. *Water Quality Engineering: Physical/Chemical Treatment Processes*; 2013.

(13) Conant, R. T.; Ryan, M. G.; Ågren, G. I.; Birge, H. E.; Davidson, E. A.; Eliasson, P. E.; Evans, S. E.; Frey, S. D.; Giardina, C. P.; Hopkins, F. M.; Hyvönen, R.; Kirschbaum, M.

U. F.; Lavallee, J. M.; Leifeld, J.; Parton, W. J.; Megan Steinweg, J.; Wallenstein, M. D.; Martin Wetterstedt, J. Å.; Bradford, M. A. Temperature and Soil Organic Matter Decomposition Rates - Synthesis of Current Knowledge and a Way Forward. *Glob. Chang. Biol.* **2011**, *17* (11), 3392–3404. https://doi.org/10.1111/j.1365-2486.2011.02496.x.
(14) DAVIDSON, E. A.; JANSSENS, I. A.; LUO, Y. On the Variability of Respiration in Terrestrial Ecosystems: Moving beyond Q 10. *Glob. Chang. Biol.* **2006**, *12* (2), 154–164. https://doi.org/10.1111/j.1365-2486.2005.01065.x.
(15) Cecchetti, A. R.; Stiegler, A. N.; Gonthier, E. A.; Bandaru, S. R. S.; Fakra, S. C.; Alvarez-Cohen, L.; Sedlak, D. L. Fate of Dissolved Nitrogen in a Horizontal Levee: Seasonal Fluctuations in Nitrate Removal Processes. *Environ. Sci. Technol.* **2022**, *56* (4), 2770–2782. https://doi.org/10.1021/acs.est.1c07512.
(16) Limmer, M. A.; Burken, J. G. Plant Translocation of Organic Compounds: Molecular and Physicochemical Predictors. *Environ. Sci. Technol. Lett.* **2014**, *1* (2), 156–161. https://doi.org/10.1021/ez400214q.
(17) Miller, E. L.; Nason, S. L.; Karthikeyan, K. G.; Pedersen, J. A. Root Uptake of Pharmaceuticals and Personal Care Product Ingredients. *Environ. Sci. Technol.* **2016**, *50* (2), 525–541. https://doi.org/10.1021/acs.est.5b01546.
(18) Shenker, M.; Harush, D.; Ben-Ari, J.; Chefetz, B. Uptake of Carbamazepine by Cucumber Plants – A Case Study Related to Irrigation with Reclaimed Wastewater. *Chemosphere* **2011**, *82* (6), 905–910. https://doi.org/10.1016/j.chemosphere.2010.10.052.
(19) McCurry, D. L.; Bear, S. E.; Bae, J.; Sedlak, D. L.; McCarty, P. L.; Mitch, W. A. Superior Removal of Disinfection Byproduct Precursors and Pharmaceuticals from Wastewater in a Staged Anaerobic Fluidized Membrane Bioreactor Compared to Activated Sludge. *Environ. Sci. Technol. Lett.* **2014**, *1* (11), 459–464. https://doi.org/10.1021/ez500279a.
(20) Bertelkamp, C.; Verliefde, A. R. D.; Schoutteten, K.; Vanhaecke, L.; Vanden Bussche, J.; Singhal, N.; van der Hoek, J. P. The Effect of Redox Conditions and Adaptation Time on Organic Micropollutant Removal during River Bank Filtration: A Laboratory-Scale Column Study. *Sci. Total Environ.* **2016**, *544*, 309–318. https://doi.org/10.1016/j.scitotenv.2015.11.035.
(21) Brienza, M.; Duwig, C.; Pérez, S.; Chiron, S. 4-Nitroso-Sulfamethoxazole Generation in Soil under Denitrifying Conditions: Field Observations versus Laboratory Results. *J. Hazard. Mater.* **2017**, *334*, 185–192. https://doi.org/10.1016/j.jhazmat.2017.04.015.
(22) Nödler, K.; Licha, T.; Barbieri, M.; Pérez, S. Evidence for the Microbially Mediated Abiotic Formation of Reversible and Non-Reversible Sulfamethoxazole Transformation Products during Denitrification. *Water Res.* **2012**, *46* (7), 2131–2139. https://doi.org/10.1016/j.watres.2012.01.028.
(23) Hübner, U.; Wurzbacher, C.; Helbling, D. E.; Drewes, J. E. Engineering of Managed Aquifer Recharge Systems to Optimize Biotransformation of Trace Organic Chemicals. *Curr. Opin. Environ. Sci. Heal.* **2022**, *27*, 100343. https://doi.org/10.1016/j.coesh.2022.100343.
(24) Harb, M.; Lou, E.; Smith, A. L.; Stadler, L. B. Perspectives on the Fate of Micropollutants in Mainstream Anaerobic Wastewater Treatment. *Curr. Opin. Biotechnol.* **2019**, *57*, 94–100. https://doi.org/10.1016/j.copbio.2019.02.022.
(25) Inyang, M.; Flowers, R.; McAvoy, D.; Dickenson, E. Biotransformation of Trace Organic Compounds by Activated Sludge from a Biological Nutrient Removal Treatment System.

Bioresour. Technol. **2016**, *216*, 778–784. https://doi.org/10.1016/j.biortech.2016.05.124.
